# Nanomedicine-Driven Modulation of the Gut–Brain Axis: Innovative Approaches to Managing Chronic Inflammation in Alzheimer’s and Parkinson’s Disease

**DOI:** 10.3390/ijms26189178

**Published:** 2025-09-19

**Authors:** Antea Krsek, Lou Marie Salomé Schleicher, Ana Jagodic, Lara Baticic

**Affiliations:** 1 Faculty of Medicine, University of Rijeka, 51000 Rijeka, Croatia; 2 Department of Family Medicine, Community Health Center Krapina, 49000 Krapina, Croatia; beganovicana05@gmail.com; 3 Department of Medical Chemistry, Biochemistry and Clinical Chemistry, Faculty of Medicine, University of Rijeka, 51000 Rijeka, Croatia

**Keywords:** Alzheimer’s disease, artificial intelligence, exposome, gut–brain axis, Parkinson’s disease

## Abstract

Chronic inflammation plays a crucial role in the pathogenesis and progression of neurodegenerative diseases such as Alzheimer’s disease (AD) and Parkinson’s disease (PD), where sustained neuroinflammatory responses contribute to neuronal damage and functional decline. Recent advances in nanomedicine offer novel therapeutic strategies aimed at modulating inflammation, with a focus on targeting the gut–brain axis, a key mediator in the interplay between systemic inflammation and neurodegeneration. Artificial intelligence (AI) has emerged as a transformative tool in this context, facilitating the integration of large, complex datasets to better understand the intricate relationship between gut microbiota dysbiosis, chronic neuroinflammation, the exposome (cumulative impact of lifelong environmental exposures), and disease manifestation. AI-driven approaches and integrating exposome data with AI enable deeper insights into exposure–microbiome–inflammation interactions, enhance our understanding of the inflammatory pathways involved, support the development of predictive models for disease progression, and optimize the delivery of nanomedicine-based therapeutics. Additionally, AI applications in neuroimaging and personalized therapy planning have shown promise in addressing both motor and non-motor symptoms. This review provides a comprehensive synthesis of current knowledge, highlighting the convergence of AI, nanomedicine, and chronic inflammation in neurodegenerative disease care.

## 1. Introduction

### 1.1. Key Facts and Players in Chronic Neuroinflammation

Chronic inflammation is increasingly recognized as a central factor in the pathogenesis and progression of neurodegenerative diseases, including Alzheimer’s disease (AD) and Parkinson’s disease (PD). In these conditions, persistent neuroinflammatory responses significantly contribute to neuronal damage, synaptic dysfunction, and ultimately to the decline of cognitive and motor functions. The sustained activation of microglia and astrocytes, along with the release of pro-inflammatory cytokines, such as interleukin-1β (IL-1β), tumor necrosis factor-α (TNF-α), and interleukin-6 (IL-6), leads to a toxic environment that exacerbates neuronal injury and accelerates disease progression [1,2,3,4].

Chronic inflammation has increasingly been acknowledged as a driver for neurodegenerative disease pathology. In AD and PD, the persistent activation of innate immunity inside the brain results in progressive neuronal injury and impaired synaptic transmission, culminating in cognitive and motor deficits [5]. Different stimuli activate and maintain this prolonged innate immune response, including the presence of protein aggregates (amyloid-beta and alpha-synuclein), oxidative stress, mitochondrial dysfunction, and damaged neuronal materials, which impart signals of danger, the so-called DAMPs (Damage-Associated Molecular Patterns). These DAMPs can engage pattern recognition receptors such as Toll-like receptors (TLRs) and NOD-like receptors (NLRs) in glial cells, with the NOD-, LRR-, and pyrin domain-containing protein 3 (NLRP3) inflammasome playing a particularly important role. This leads to an exacerbative cycle of pro-inflammatory cytokines and chemokines release, particularly IL-1β, IL-6, and TNF-α, which increase microglial reactivity and disrupt neuronal homeostasis [6]. This chronic neuroinflammatory status has compounded the effects of aging-related changes in immune regulation, including impaired autophagy, decreased anti-inflammatory cytokine production (such as IL-10), and defective clearance of inflammatory mediators [7]. The heavy inflammatory load is not restricted to the CNS, though know this: peripheral inflammation with origins in gut dysbiosis or systemic infections primes CNS immune responses by lowering the activation threshold of microglia, thereby potentiating neurodegenerative processes. Hence, an understanding of and therapeutic targeting of chronic inflammation, both central and peripheral, becomes important in the creation of disease-modifying treatments for AD and PD [8].

Growing scientific evidence highlights the key role of the gut–brain axis (GBA) in modulating neuroinflammation. The gut microbiota, through the production of metabolites, immune modulation, and direct neural communication, can influence central nervous system (CNS) homeostasis. Dysbiosis, characterized by an imbalance in gut microbial composition, has been associated with increased intestinal permeability and translocation of endotoxins, such as lipopolysaccharide (LPS), into systemic circulation. This phenomenon can trigger systemic inflammation, subsequently affecting the CNS through microglial activation and the disruption of the blood–brain barrier (BBB) [9,10].

**Figure 1:**
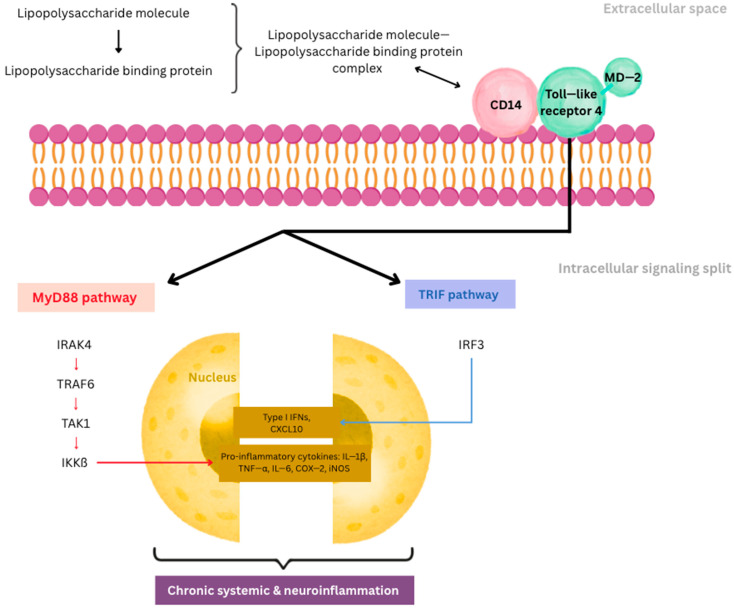
Pathways of LPS-TLR4 signaling in sustaining systemic and neuroinflammation. IRAK4—interleukin-1 receptor-associated kinase 4; TRAF6—tumor necrosis factor receptor-associated factor 6; TAK1—transforming growth factor-beta-activated kinase 1; IKKβ—inhibitory kappa B kinase beta; IRF3—interferon regulatory factor 3.

LPS, a potent pro-inflammatory glycolipid derived from the outer membrane of Gram-negative bacteria, plays a critical role in sustaining chronic systemic and neuroinflammation. Once LPS enters systemic circulation—typically via translocation across a compromised intestinal barrier—it binds to LPS-binding protein (LBP) in the plasma [11]. This complex then transfers LPS to membrane-bound CD14 (mCD14) on the surface of innate immune cells such as monocytes, macrophages, and dendritic cells, forming a tri-molecular signaling complex with Toll-like receptor 4 (TLR4) and its co-receptor MD-2 [12]. The LPS-TLR4-MD2 interaction triggers a dual intracellular signaling cascade: the MyD88-dependent and the TRIF-dependent pathways. The MyD88 pathway leads to rapid activation of NF-κB and AP-1 transcription factors through a kinase cascade involving IRAK4, TRAF6, TAK1, and IKKβ. This results in the transcriptional upregulation of pro-inflammatory genes, including IL-1β, TNF-α, IL-6, COX-2, and inducible nitric oxide synthase (iNOS) [13]. Simultaneously, the TRIF pathway activates IRF3, which induces type I interferons and additional chemokines such as CXCL10, further expanding the inflammatory response. LPS also induces metabolic reprogramming of immune cells—a process termed immunometabolism—favoring aerobic glycolysis (Warburg effect) over oxidative phosphorylation, which supports sustained cytokine production and a pro-inflammatory macrophage phenotype (M1 polarization). This shift enhances the cell’s inflammatory capacity and perpetuates the chronic state of immune activation as presented on Figure 1 [14].

Systemically, LPS-driven inflammation leads to the production of circulating cytokines and acute-phase proteins such as C-reactive protein (CRP) and serum amyloid A, which affect endothelial function, insulin signaling, and neurovascular integrity. Prolonged exposure to LPS has been shown to increase BBB permeability by downregulating tight junction proteins (e.g., claudin-5 and occludin) in brain endothelial cells and by promoting endothelial oxidative stress through NADPH oxidase and mitochondrial reactive oxygen species (ROS) production [15].

Within the CNS, LPS either crosses the compromised BBB or signals via vagal afferents to activate the hypothalamus and brainstem inflammatory circuits. In the brain parenchyma, LPS stimulates microglial TLR4, promoting their transition to a reactive state characterized by enhanced production of ROS, TNF-α, IL-1β, and prostaglandins, often accompanied by the formation of the NLRP3 inflammasome. The resulting chronic neuroinflammatory state disrupts synaptic transmission, contributes to dendritic spine loss, and enhances the phosphorylation and aggregation of tau and alpha-synuclein—core pathological processes in Alzheimer’s and Parkinson’s disease, respectively [16]. Persistent low-dose LPS exposure, as seen in chronic gut leakage, subtly maintains a low-grade inflammatory tone systemically and centrally. This steady-state activation is sufficient to drive oxidative stress, mitochondrial dysfunction, and impaired clearance of neurotoxic proteins, leading to progressive neuronal injury over time [17]. LPS operates as a molecular bridge between peripheral and central inflammation. Its ability to engage innate immune receptors, reprogram cellular metabolism, compromise the BBB, and chronically activate microglia underlies its pivotal role in inflammaging and the pathogenesis of neurodegenerative diseases [18].

Advancements in nanomedicine have introduced innovative therapeutic strategies aimed at regulating neuroinflammation by targeting both central and peripheral inflammatory pathways. Nanoparticles designed for precise delivery of anti-inflammatory agents can cross the BBB and interact directly with neuroinflammatory foci, offering localized therapeutic effects with minimal systemic exposure. Additionally, targeted modulation of the gut microbiota using functionalized nanoparticles offers a promising approach to restoring gut–brain homeostasis. By delivering prebiotics, probiotics, or anti-inflammatory molecules specifically to dysbiotic regions, these nanosystems can reduce systemic inflammation and indirectly attenuate neuroinflammatory processes. The integration of nanotechnology with a deeper understanding of the GBA thus holds significant potential for developing more effective therapeutic interventions for neurodegenerative diseases, addressing inflammation both at its central source and peripheral triggers [19,20].

In recent years, artificial intelligence (AI) has emerged as a transformative medical tool with the potential to revolutionize our understanding, diagnosis, and treatment of neurodegenerative diseases. AI can spot patterns and insights that are usually too complex to be noticed by other methods using large datasets, sophisticated algorithms, and machine learning techniques. In the context of AD and PD, AI holds promise for early detection, prediction of disease progression, and the development of personalized therapeutic strategies [1,2,3,19].

AD and PD are a rising global health burden characterized by progressive cognitive decline and motor dysfunction and have a significant impact on the quality of life. Despite decades of research, these disorders’ complex etiology and pathophysiology remain incompletely understood, complicating efforts to develop effective diagnostic and therapeutic strategies. Current approaches mainly aim at symptom management, with limited success in altering the disease course, and underscore the urgent need for new approaches to diagnosis and treatment [4,9]. The emerging recognition of the microbiome’s influence on neurodegenerative diseases, coupled with AI’s capacity to unravel complex biological networks, offers a unique opportunity to explore how dysbiosis contributes to disease onset and progression, paving the way for innovative diagnostic and therapeutic strategy targeting (GBA) [10]. AI opens new dimensions toward mapping complex interplays between the microbiome and brain health in this context. AI analysis of big data could identify biomarkers of dysbiosis and model disease progression, thus supporting personalized treatment approaches along the GBA [19]. This review aimed to provide a comprehensive synthesis of the current knowledge in this rapidly evolving and highly dynamic scientific field.

### 1.2. Molecular Mediators of Chronic Neuroinflammation

Neuroinflammation in chronic neurodegenerative diseases extends beyond a simple reaction to protein accumulations such as amyloid-beta or alpha-synuclein; it reflects a prolonged and interconnected disruption of immune signaling, cellular metabolism, and structural integrity within the brain. While the activation of microglia and astrocytes is well documented, less attention is often given to the non-cell autonomous propagation of inflammation, where signaling molecules released by one glial subtype influence others and even distant brain regions via cytokine gradients and extracellular vesicles [21].

In the initial immune response, ATP, mitochondrial DNA, and oxidized lipids released from stressed neurons—serve as danger-associated molecular patterns (DAMPs) that stimulate purinergic receptors like P2X7 on microglia. P2X7 engagement not only promotes NLRP3 inflammasome assembly but also induces K^+^ efflux, a key biochemical step that licenses inflammasome activation. Importantly, this ionic imbalance also perturbs calcium signaling, mitochondrial potential, and ROS production, reinforcing the inflammatory state [22].

Astrocytes, beyond undergoing reactive astrogliosis, play a critical but often underexplored role in modulating the synaptic environment through glutamate homeostasis. Under chronic inflammatory conditions, their reduced expression of EAAT1/EAAT2 (glutamate transporters) leads to synaptic glutamate spillover, excitotoxicity, and postsynaptic calcium overload. This causes neuronal apoptosis via activation of calpain and caspase cascades, especially in glutamate-sensitive neurons of the hippocampus and substantia nigra [23].

The NLRP3 inflammasome, while central to IL-1β and IL-18 release, also intersects with autophagy dysfunction, a less detailed area. Normally, autophagy regulates inflammasome turnover by degrading NLRP3 complexes via p62-mediated ubiquitination and lysosomal trafficking. However, chronic activation of NLRP3 impairs this feedback by altering lysosomal pH and rupturing lysosomal membranes, releasing cathepsin B, which paradoxically feeds back to promote more inflammasome activation. This creates a vicious cycle of uncontrolled inflammation and impaired clearance of toxic aggregates [24].

Another potent amplifier is HMGB1, which can bind not only to TLR4 but also to RAGE (Receptor for Advanced Glycation End Products). HMGB1-RAGE signaling contributes to sustained ERK and p38 MAPK activation, leading to downstream NF-κB transcriptional activity and epigenetic modifications that favor a pro-inflammatory microglial phenotype [25]. Furthermore, HMGB1 exists in different redox states—fully reduced, disulfide, and oxidized—each of which has distinct receptor affinities and bioactivities. The disulfide form, in particular, is associated with cytokine-inducing potential and is elevated in CSF of patients with AD and PD [26]. While NF-κB activation is a canonical pathway in inflammation, its chronic activation also modifies glial cell metabolism. Long-term NF-κB signaling shifts microglial energetics from oxidative phosphorylation toward aerobic glycolysis (Warburg-like effect), increasing lactate production and acidifying the extracellular environment, which further damages myelin and axons. This metabolic remodeling impairs mitochondrial biogenesis and ATP synthesis, key requirements for effective neuronal repair [27].

Finally, a critically underexplored mechanism in the resolution phase of inflammation is the role of specialized pro-resolving mediators (SPMs)—including resolvins (RvD1 and RvE1), lipoxins, maresins, and protectins. These lipid-derived molecules are biosynthesized from omega-3 fatty acids via lipoxygenase (ALOX) enzymes, and they act on specific G-protein-coupled receptors (e.g., ALX/FPR2 and ChemR23) to limit neutrophil infiltration, enhance efferocytosis of apoptotic cells, and restore tissue homeostasis [28]. In aging and neurodegenerative conditions, SPM levels are significantly reduced, and their biosynthetic enzymes are downregulated, leading to failed resolution of inflammation. This lack of resolution is not passive—it actively contributes to prolonged microglial activation and neuronal vulnerability [29]. All together, these under-addressed elements—ionic flux, astrocytic glutamate handling, inflammasome-autophagy cross-talk, HMGB1-RAGE redox signaling, metabolic reprogramming, and impaired pro-resolving mediator synthesis—construct a biological landscape of chronic immune activation that is both cause and consequence of neurodegeneration [30]. Table 1 summarizes molecular and systemic drivers of chronic neuroinflammation in AD and PD.

## 2. Gut–Brain Interactions

The GBA, a bidirectional communication network linking the CNS with the GI, has emerged as a critical player in the pathophysiology of neurodegenerative diseases. Increasing knowledge indicates that alterations in gut microbiota composition and intestinal barrier function can modulate neuroinflammation, protein aggregation, and neuronal survival. Microbial metabolites, immune signaling, and vagal nerve pathways represent key mediators of this cross-talk, especially in AD and PD [10,16,18,21]. Understanding the mechanisms of gut–brain interactions offers new perspectives on disease onset and progression and may open novel avenues for therapeutic intervention targeting the microbiome.

### 2.1. Artificial Intelligence in Mapping Gut–Brain Interactions

Major applications of AI in research on gut microbiomes are seen in analyses related to composition and diversity [20]. AI in biomedical sciences generally provides the tools and techniques required to gather, organize, and analyze big biological data, including nutritional, genomic, and associated information [31]. AI systems will process high-throughput sequencing data to identify and quantify the different microbial species in the gut. The interactions between gut microbiota and their hosts are even better known, as such an approach conveys an integral vision of human metabolism [32,33]. Artificial intelligence diagnostics use sophisticated algorithms that analyze various data sets, including stool samples, blood tests, and case histories [34]. AI-enabled tools find their applications in neuroscience for decoding brain signals, mapping neural networks, and analyzing large-scale neuroimaging data. Machine learning algorithms identify brain activity patterns correlating with conditions such as Alzheimer’s and Parkinson’s. These patterns, once recognized, have helped AI models contribute toward early diagnosis, personalized treatment planning, and the prediction of disease progression [2]. AI also furthers, in a significant manner, the development of brain–computer Interfaces (BCIs) that are used to control prosthetics and communicative devices with greater precision, thus giving rise to improvements in the lives of people with neurodegenerative diseases.

This capability, powered by advanced computational tools, profiles individual people and population microbiomes, enabling resolutions and completions previously unattainable. In microbiome research, AI analyzes genomic and metagenomic data produced by microbial communities, especially gut-dwelling [35]. The gut microbiome is intermingled with brain health, behavior, cognition, and neurodevelopment. AI-driven models help decipher the complex relationships of microbiome composition with neurological health, furthering our knowledge of how gut microbes influence different neurological conditions [34]. Further, AI algorithms have been employed to analyze interactions of gut microbes with host physiology in an attempt to disclose the mechanisms that drive changes in calorie intake and metabolic disposition. AI in biomedical nutrition research meets the dire need to engage in effective analysis and interpretation of nutrition’s highly interdependent relationship with human physiology, especially concerning the gut microbiome [36,37].

### 2.2. Analytical Tools and Technologies for AI in Mapping Gut–Brain Interactions

To address the complexity of gut–brain communication, AI research increasingly relies on specialized analytical platforms and computational technologies. Deep learning frameworks, such as convolutional neural networks and recurrent neural networks, are used to extract patterns from high-dimensional biological datasets, including metagenomic sequencing and functional neuroimaging [2,3]. Natural language processing (NLP) techniques are employed to mine the biomedical literature and clinical records, helping to identify emerging associations between microbial taxa and neurological phenotypes [33,34,35,36]. Multi-omics integration platforms powered by AI enable the combined analysis of genomics, metabolomics, transcriptomics, and proteomics data, offering a systems-level perspective on host–microbiome–brain interactions [37]. Together, these technologies form the foundation for understanding the complex two-way interactions between the gut and brain in neurodegenerative diseases.

## 3. Alzheimer’s Disease (AD)

AD is a progressive neurological disorder and the leading cause of dementia, characterized by memory loss, cognitive decline, and impaired daily functioning [38]. Hallmarked by brain plaques and tangles, AD progresses through mild, moderate, and severe stages, with early symptoms often mistaken for normal aging. Early diagnosis, critical for effective symptom management and slowing disease progression, relies on cognitive assessments, neuroimaging, and genetic testing [39,40]. Artificial intelligence has contributed much to medicine in general and neuroimaging and diagnostics related to AD. Artificial intelligence will revolutionize healthcare by applying insights derived from data to diagnostics, treatment planning, and the development of new medications. These AI algorithms analyze a large volume of medical data to facilitate the early detection of diseases through image analysis and predictive analytics [41]. It enhances diagnostic precision and helps optimize treatment strategies to suit the needs of individual patients.

These research questions are identified at the core of improvements in AI-based ADD (Alzheimer’s disease detection). The importance of understanding available imaging techniques lays the basis for choosing appropriate methods, and being aware of common pre-processing methods helps to ensure that high-quality data are maintained. Segmentation methods identify pathological areas within the image for detailed analysis, and optimization techniques work to enhance image clarity [39]. Familiarity with various learning algorithms strengthens an AI model for more accurate detection of AD, hence creating possibilities for earlier diagnosis and management. Medical imaging and neuroimaging are fast-growing fields, and their development is supported by continuous technological advances, new computational techniques, and artificial intelligence [42].

### AI Mapping of Gut–Brain Interactions in Alzheimer’s Disease

Recent developments in the study of AD have started to reveal the complex interactions between the body and brain. The GBA is one of the most exciting new fields, indicating that there may be a close connection between the health of the gastrointestinal tract and how well the brain works. Because of the linking of those two fields, researchers are looking into the potential influence of the billions of microorganisms in the gut on brain health and illness. Artificial intelligence has become an essential tool in this area of research and offers new approaches to the analysis of biological data and the discovery of previously unnoticed patterns of neurodegenerative illnesses like Alzheimer’s.

Numerous microorganisms, including bacteria, fungi, and viruses, live in the gastrointestinal (GI) tract. These organisms are generally referred to as the gut microbiota (sometimes used interchangeably with “microbiome”, which refers to the collection of genomes from all microorganisms) [43]. Through a variety of pathways controlling peripheral neurotransmitters, metabolites, and immunological signaling molecules, dysbiosis—an unbalanced population of gut microbiota—has been connected to some brain disorders, according to recently mounting evidence [44,45].

Early AD identification is difficult since symptoms do not appear until a considerable number of neurons have been destroyed. As a result, early management is challenging because conventional diagnostic techniques frequently miss the disease in its early stages [46]. This gap may be filled in part by recent developments in AI, especially in machine learning techniques. Early detection attempts have substantially benefited from the analysis of magnetic resonance imaging (MRI) images with the use of machine learning techniques.

Support vector machines (SVMs) have been demonstrated to be effective in evaluating MRI scans and distinguishing between patients with AD, frontotemporal lobar degeneration (FTLD), and healthy controls. To improve diagnostic precision, more research has focused on the identification of AD using 3D neural network architectures [47].

A special emphasis has been placed on patients with mild cognitive impairment (MCI), who are at a high risk of acquiring Alzheimer’s. For example, up to three years before a clinical diagnosis can be made, random forest algorithms have been used to forecast the change from MCI to AD. Furthermore, to identify AD and related dementias, complete models utilizing a variety of machine learning techniques, such as Naïve Bayesian, decision trees, and SVMs, have been used. These studies demonstrate how AI could transform AD by facilitating earlier and more accurate detection. These AI-powered methods not only improve the understanding of the causes and progression of the disease but also open the door to timely and targeted interventions that may revolutionize the way AD is managed [48].

## 4. Parkinson’s Disease

Neurodegenerative diseases like PD pose significant challenges to the healthcare system, affecting over 50 million individuals and resulting in an economic burden exceeding USD 300 billion [9]. Parkinson’s disease, in particular, is recognized as the fastest-growing neurodegenerative disorder globally, with its prevalence increasing at a rate comparable to a pandemic [49]. Recent statistics indicate that 11.8 million people around the world are currently living with PD [50]. For example, in PD, treatments like L-dopa result in symptomatic relief, whereas active investigations continue in a search for disease-modifying therapies, with over sixty current clinical trials testing potential drugs in addition to complementary studies examining lifestyle interventions [51,52].

The diversity of pharmacological approaches under investigation (ranging from small molecules, biologics, cell, and gene therapies to anti-inflammatories) reflects the complexity of the disease process, which is typified by multivariate pathophysiology, significant phenotypic heterogeneity, and prodromal phases that may precede the appearance of clinically identifiable motor and non-motor symptoms by several decades [53,54]. Recently, there have been growing demands for the biological redefinition of Parkinson’s based on genetic and biomarker classification systems [55,56,57]. For example, much of the PD spectrum and related disorders might be classified as “Neuronal alpha-synuclein Disease” (NSD), which would encompass diseases such as dementia with Lewy bodies characterized by distinctive Lewy body and Lewy neurite pathology in neurons of both the central and peripheral nervous systems [55,56]. The absence of validated disease progression markers further complicates the task of designing and assessing drugs that could alleviate, mitigate, or even reverse symptoms. Identification of metrics that more precisely detect early changes in disease would facilitate drug development in PD by enhancing the evaluation of therapeutic agents for efficacy and clinical effectiveness. Besides digital monitoring technologies, substantial developments in the federated use of AI and Machine Learning (ML) could take this effort considerably further, building on foundational frameworks [58,59,60].

In parallel with advances in genetic and digital biomarker classification, accumulating evidence highlights chronic neuroinflammation as a central pathophysiological mechanism in Parkinson’s disease, with direct implications for both diagnosis and therapeutic targeting. While dopaminergic neurodegeneration has long been the clinical hallmark of PD, current research shows that this neuronal loss occurs within a broader context of sustained immune dysregulation affecting both the central and peripheral nervous systems [61]. Aggregated alpha-synuclein—particularly its misfolded and phosphorylated forms—acts not only as a pathological hallmark but also as a pro-inflammatory stimulus, engaging innate immune receptors such as TLR2, TLR4, and scavenger receptors on microglia. This leads to downstream activation of NF-κB signaling, production of ROS and RNS, and the release of cytokines that potentiate oxidative stress and direct neurotoxicity in vulnerable dopaminergic circuits. Beyond microglia, astrocytic dysfunction and microglial senescence contribute to impaired glutamate clearance, trophic support loss, and ineffective α-synuclein clearance—exacerbating the neuroinflammatory milieu [62].

Emerging studies also reveal the infiltration of adaptive immune cells into the substantia nigra, particularly CD4^+^ Th17 and CD8^+^ T cells, which secrete cytotoxic mediators such as granzyme B and IFN-γ, contributing to neuronal injury and sustaining local inflammation. This is often accompanied by BBB disruption, allowing peripheral cytokines, immune cells, and microbial byproducts to gain access to the CNS. Importantly, PD-associated inflammation is not restricted to the brain [63]. A growing body of research supports a “gut-first” hypothesis, wherein alpha-synuclein pathology originates in the enteric nervous system—potentially triggered by gut microbial dysbiosis or exposure to bacterial amyloids like curli—and ascends to the brain via the vagus nerve. This is supported by the early presence of α-synuclein aggregates in the gastrointestinal tract and vagal nuclei in prodromal PD. Microbial metabolites, such as short-chain fatty acids, hydrogen sulfide, and LPS, modulate enteric immune activity and may initiate both local and systemic inflammation [64].

At the systemic level, inflammatory mediators such as IL-6, CRP, and TNF-α not only mirror central pathology but also correlate with motor symptom severity and progression, while extracellular vesicles and complement proteins (e.g., C1q and C3b) are emerging as dynamic vehicles of neuroimmune signaling and potential diagnostic indicators [65]. Despite these insights, resolution pathways—normally regulated by lipid-derived molecules such as resolvins, protectins, and lipoxins—appear to be defective in PD, leading to a failure in terminating inflammation and restoring homeostasis. Together, these findings highlight a paradigm shift in our understanding of PD, positioning inflammation as both a driver of pathogenesis and a promising therapeutic target [66]. They also underscore the potential of AI-augmented multiomic biomarker platforms to capture the heterogeneity of inflammatory profiles across individuals. Integrating immune-related metrics into disease stratification frameworks could profoundly enhance the personalization of treatment strategies and support the development of disease-modifying therapies aimed at interrupting the inflammatory cascade early in its course [67].

### Robot-Assisted Gait Training and AI Applications in Parkinson’s Disease

Gait freezing is one of the most common and debilitating motor symptoms in PD patients, significantly impacting patients’ mobility and quality of life. Robot-assisted gait training (RAGT) has recently emerged as a promising therapeutic approach for gait rehabilitation, showing advantages over traditional methods [68]. Studies indicate that RAGT improves gait kinematics and spatiotemporal parameters and may be effective even in patients who have previously received treatment with deep-brain stimulation (DBS) [69,70]. Comparative analyses reveal RAGT’s superiority in enhancing motor learning and reducing freezing episodes, although parameters like body weight support and guidance force require further refinement [71,72]. The first quantitative comparative analysis between RAGT and treadmill training in PD showed that, after robotic training, significant improvements in gait kinematics were evident mainly in the frontal plane at the pelvic and hip levels [73]. Kang et al. further investigated the effects of RAGT with the use of Walkbot-S^TM^ on changes in gait velocity and brain functional networks, emphasizing its role in enhancing gait automaticity [74]. Another study pointed out that RAGT was superior to treadmill training in reducing gait freezing episodes and improving walking endurance and suggested that medium- and long-term follow-ups could be performed to ensure better retention of motor learning and sustained benefits [75].

AI-driven solutions significantly enhance the prediction and management of gait freezing in PD. The binary classification algorithms showed better performance compared to traditional three-class prediction models, offering a more reliable method for anticipating freezing episodes and thus timely interventions [76]. Convolutional neural networks analyzing plantar pressure data represented as 2D images further contribute to the development of real-time detection, thus allowing for proactive management of gait freezing [77].

Long-term RAGT studies have also shown the possibility of long-term balance and gait improvements in persons with PD. Bevilacqua et al. underscored that the long-lasting effect of RAGT is reflected in the motor improvement at six months, one year, and two years after rehabilitation in older adults with PD. These findings point out the importance of extended follow-ups to confirm the retention of motor learning and the effectiveness of robotic interventions in the treatment of gait abnormalities [78].

Besides the management of motor symptoms, AI applications drive innovation in drug discovery for PD. Techniques such as Weighted Gene Co-expression Network Analysis (WGCNA) enable the identification of key dysregulated pathways and modules associated with the disease. It can lead to an approach that can more easily introduce new drug candidates that will be able to restore system homeostasis to bring hope to poor responders of conventional treatments, such as levodopa and deep brain stimulation [79]. It opens not only a route to personalized treatment strategies in PD management but also a wide door for a bright future in the integration of robotic systems with AI technologies great leap toward precision medicine. Table 2 and Table 3 summarize computational approaches for AD and PD, emphasizing their advantages and disadvantages.

## 5. Imaging in AI

AI can integrate multi-omics, imaging, and clinical datasets to model the cascade linking systemic inflammation to CNS pathology. By analyzing high-dimensional data, AI algorithms can detect biomarkers of BBB disruption, map neuroinflammatory signaling pathways, and correlate these with structural and functional brain changes. Machine learning models reveal how inflammatory mediators alter intracellular signaling (e.g., kinase activation and oxidative stress) and intercellular communication (e.g., cytokine–glia interactions), thereby connecting peripheral immune activation to neuronal damage. In this way, AI enables a systems-level understanding of how systemic inflammation drives BBB dysfunction, neuroinflammation, and progressive neurological injury [53,77].

Biomarkers associated with Alzheimer’s dementia need to be analyzed using imaging techniques such as MRI, positron emission tomography (PET), and electroencephalography (EEG) for early detection and to understand the disease mechanism. MRI helps in capturing structural changes in the brain, including atrophy and regional changes, which could help in identifying potential biomarkers [80]. PET scans are important for the detection of molecular changes, especially abnormal protein deposits such as beta-amyloid plaques and tau tangles, which are considered hallmarks of AD [99]. EEG offers functional information by measuring the electrical activity of the brain, detecting abnormalities, and assessing connectivity disruptions. These modalities—MRI, PET, and EEG—are very vital for the diagnosis of AD and PD at an early stage [100]. The process involves neuroimage pre-processing, segmentation, and feature extraction, followed by classification and prediction that support early diagnosis of Alzheimer’s dementia [101,102,103]. This broad approach lets there be better diagnostic accuracy and may thus lead to earlier interventions. The integration of structural and functional biomarkers offers a holistic view of Alzheimer’s-related changes, improving diagnostic precision [104]. The Alzheimer’s Disease Neuroimaging Initiative (ADNI) involves longitudinal data from ADNI-1, ADNI-2, and ADNI-3, which track clinical, imaging, genetic, and biochemical information across various stages of Alzheimer’s, including mild cognitive impairment and healthy controls [105].

These datasets are very useful for understanding disease progression and biomarker identification and assessing treatment responses. Moreover, the Open Access Series of Imaging Studies (OASIS) gives cross-sectional MRI data on Alzheimer’s patients, MCI cases, and healthy controls, thus offering a valuable resource for studying neurodegenerative structural changes [106].

**Figure 2:**
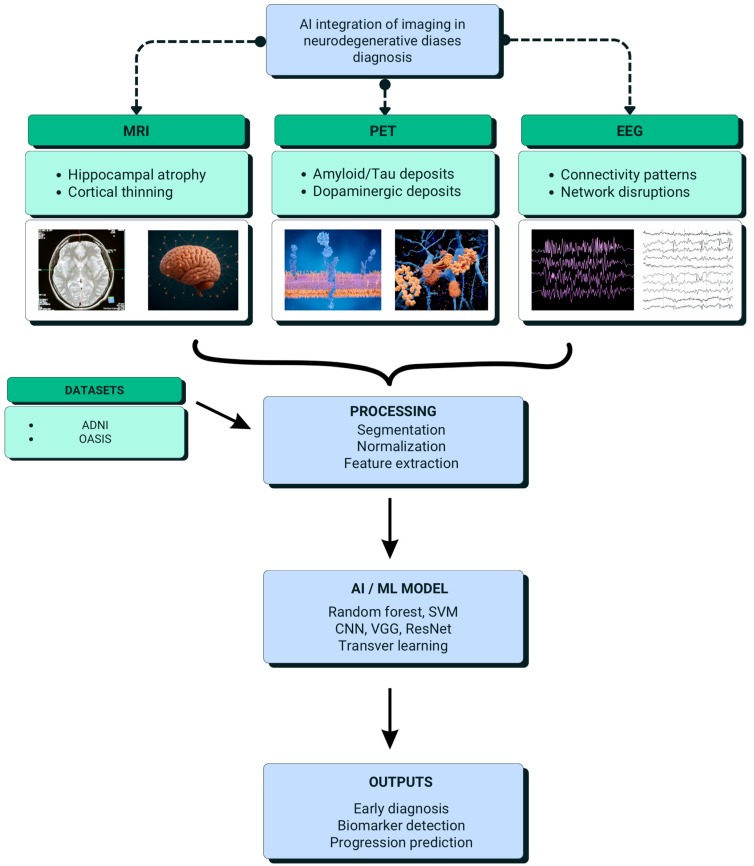
AI integration of multimodal imaging in neurodegenerative disease diagnosis. MRI, PET, and EEG provide structural, molecular, and functional biomarkers that undergo preprocessing and feature extraction before being analyzed by AI/ML models such as random forest, SVM, and deep learning networks (CNN, ResNet, and VGG). Large imaging datasets (ADNI and OASIS) supply data for model training and validation. The outputs include biomarker detection, early diagnosis, disease stratification, and prediction of disease progression.

Artificial Bee Colony (ABC) algorithms, inspired by honeybee foraging, are employed to optimize the selection of critical features or weights in machine learning models, thereby improving AD classification and prediction. In one study, brain image segmentation was carried out using a multi-level thresholding approach, initially applying Particle Swarm Optimization (PSO) and then enhancing it with a Markov Random Field model [107]. Another study examined the segmentation of the hippocampal region from brain subregions using various optimization techniques, including the Lion Optimization Algorithm (LOA), Genetic Algorithm (GA), BAT algorithm, Particle Swarm Optimization (PSO), and Artificial Bee Colony (ABC) optimization. The LOA outperformed the others due to its ability to avoid local optima [81]. Additionally, a hybrid approach combining GA and PSO with a deep neural network was proposed for more efficient disease classification using brain MRI images [82]. Figure 2 illustrates AI integration of multimodal imaging in neurodegenerative disease diagnosis.

## 6. Types of AI Algorithms Used in Microbiome Research

Integrating artificial intelligence into the study of microbiome–gut–brain has greatly improved knowledge of these complex interactions. AI algorithms are good at processing data, classifying it, and making predictions, hence their necessity in this field.

### 6.1. Machine Learning in Microbiome Research

Machine learning, a subfield of AI, develops algorithms and statistical models that allow computers to learn from data and improve performance in specific tasks without requiring explicit programming. It uses methods from statistics to provide machines with the capability of finding patterns and making predictions, as well as optimizing decision-making and identifying patterns based on imaging data [83]. These algorithms learn from data by identifying patterns within it and generalizing their findings, making them capable of predicting or deciding over any new, unseen dataset. Various ML algorithms are specialized for specific tasks. Supervised learning algorithms normally operate on labeled data, where input data are tagged or coupled to corresponding output labels, to predict future outputs accurately. In machine learning, the best model is usually chosen after experiments and tuning [84]. In the area of microbiome research, ML is indispensable on many levels. Hence, most neuroimaging studies have focused on advanced ML algorithms that might help distinguish AD and normal cognition [85].

The prior ML work with a basis of structural MRI has been able to demonstrate efficiency in classifying subjects along the AD continuum, from cognitively normal adults to MCI and AD patients [86,87]. Most previous neuroimaging studies using ML have relied on single classifiers such as support vector machines and linear discriminant analysis [88]. Ensemble support vector machine classification of dementia uses structural MRI and mini-mental state examination (MMSE) [89].

Additionally, Gradient Boosting Machines (GBMs), an ensemble learning method that generates models in a greedy stage-wise fashion. Each iteration of the model corrects the errors of its predecessor, thereby improving the accuracy of predictions. For microbiome research, the GBM will be able to develop predictive models of health outcomes from microbial composition and assess the important relationship with human health. GBMs are also efficient in dealing with microbiome data of a heterogeneous nature, with both categorical and numerical features [108].

Recent advances in diagnostic technologies finally enabled the assessment of AD pathologies using in vivo biomarkers, which coincided with post-mortem findings and provided important diagnostic tools [109]. The National Institute on Aging and Alzheimer’s Association introduced the amyloid-β, tau, and neurodegeneration (ATN) system for defining and staging AD based on three key biomarkers: amyloid-β, pathologic tau, and neurodegeneration [110]. Neurodegenerative changes may precede symptoms, so biomarkers such as reduced hippocampal volume and decreased cortical thickness detected through structural MRI have become increasingly useful in the stratification of AD progression [111,112]. Several neuroimaging initiatives with large, accessible databases have driven automated whole-brain pattern recognition approaches in the early detection of AD [113].

Before ML-based diagnostic tools can be put to general clinical use, several challenges remain. First, the models need to produce stable diagnostic results independent of variations in MRI scanners, magnetic field strength, image resolution, or pulse sequences [114,115]. They should provide reliable results in a mixed population regarding age, sex, and race. Therefore, more studies with more participants and the use of the data from older 1.5T MRIs and advanced MRIs, such as 3T scanners, are needed for increased clinical applications of ML-based diagnostic systems [114,116].

#### 6.1.1. Transfer Learning in Alzheimer’s Disease Detection

The concept of transfer learning has lately become pivotal in machine learning, showing great advances in the diagnosis of AD. It relies on pre-trained deep learning models, fine-tuned for optimum performance in specialized tasks such as the analysis of medical imaging data, PET, and EEG to identify AD. The most common architectures used are Residual Network (ResNet), Visual Geometry Group (VGG16 and VGG19), AlexNet, MobileNet, InceptionV3, InceptionV4, DenseNet, and LeNet [117,118]. ResNet, which was popularized by its residual learning for training deep networks, has also been very successful in AD classification [119,120]. VGG16 and VGG19 models have gained recognition due to their depth and simplicity, performing very well in image-based tasks related to AD detection. AlexNet, an initial model in deep learning, made many improvements in image classification [41,120].

In the detection of AD, inception modules have also been used by InceptionV3 and InceptionV4 to optimize their computational efficiency [121,122,123]. These pre-trained models can be further fine-tuned on AD-specific datasets through transfer learning, enabling a more accurate and computationally efficient diagnostic process that offers hope for early intervention. While ResNet-18 has been studied in depth, DenseNet-121 outperformed other architectures for the diagnosis of AD [121,124]. Another work used InceptionV3 on an international AD dataset of brain MRI images, while another work fine-tuned AlexNet for AD image classification [123,125].

Other works have explored the combination of 3D-DenseNets with different probability-based fusion methods and have carried out extensive hyperparameter tuning to improve performance [126]. The deep multi-task multi-channel learning (DM2L) framework integrates MRI data with demographic information, enabling disease classification and clinical score regression simultaneously [127]. Another approach combined global and local features using 3D densely connected convolutional networks with shape analysis for AD diagnosis [128]. A multi-task CNN using DenseNet features was also proposed to improve the effectiveness of classification [95].

AlexNet, pre-trained on ImageNet, has also been applied in complex classification applications. ResNet-29 as an end-to-end 3D-CNN was the result of a training process from transfer learning that utilized sMRI scans as inputs [129,130]. Deep convolutional neural networks such as VGG16/VGG19 pre-trained on MRI datasets through transfer learning boost AD image classification performance [131]. VoxCNNs and a random forest classifier have independently performed four-class classification tasks [132]. A hybrid model that integrates the convolutional and recurrent neural network for hippocampus analysis was also proposed [133]. Fine-tuning of AlexNet was also shown to be promising for image classification tasks [134].

Some ensemble learning approaches that combine multiple deep learning networks in volumetric and grid-based feature evaluation of brain scans have reported a diagnostic accuracy as high as 91.83% for detecting AD [135,136]. Multimodal imaging, extracting structural and functional features from both MRI and FDG-PET by deep neural networks, outperforms the single-modality best models for the diagnosis of dementia [137]. With the integration of multisource data, a deep ensemble learning framework outperforms six ensemble methods of state-of-the-art methods, improving 4% and achieving 91.54% accuracy on the OASIS dataset by integrating EfficientNetV2S-based transfer learning with densely learned features [138]. A new ensemble deep neural network, MultiAz-Net, has been developed to incorporate PET and MRI data for further enhancement in AD diagnostic capability [96].

A comprehensive study was conducted on 14 commonly used transfer learning models for AD classification and proposed high-performance models like InceptionV3, ResNet101, DenseNet121, and EfficientNetB7 [97]. Models like InceptionV3 and DenseNet201 have been optimized using variance-based pruning and Average Top-K (Avg-TopK) pooling to enhance their efficiency [98]. The MTAP model implemented with ADASYN, pruning, and Avg-TopK pooling allowed the features to be effectively extracted and gave a maximum accuracy of 99.69% [139]. Finally, there is the RGB-Angle-Wheel data augmentation approach that includes the rotation of color channels, enhancing the accuracy and robustness of deep learning models on image-processing tasks [140].

#### 6.1.2. Tau and Tubulin

Among the dual-specificity tyrosine (Y)-phosphorylation-regulated kinase (*DYRK*) family, *DYRK1A* has been given considerable attention concerning neurological diseases. This gene is highly conserved and also found in mice and rats; it is an important gene in neurological development [141]. More precisely, *DYRK1A* is involved in the generation and maturation of dendritic cells in the central nervous system, which is responsible for the growth and development of the brain [142]. It influences multiple signaling pathways by interacting with transcription factors, splicing factors, and cell-cycle-related proteins [143]. *DYRK1A* dysregulation is implicated in neurodegenerative diseases, notably Down syndrome and AD [144].

Tau and tubulin, two interdependent proteins, are crucial for maintaining axonal structure and intracellular transport [145]. The stability of tubulin is highly regulated by post-translational modifications, with phosphorylation being one of the most important. Abnormal *DYRK1A* activity has been demonstrated to hyperphosphorylated tau at key sites, that is, S202, S404, and T212, which are associated with the formation of neurofibrillary tangles (NFTs). Increased phosphorylated tau levels in *DYRK1A* transgenic mice were associated with memory deficits. Moreover, dysregulation of the expression of *DYRK1A* has also been related to certain tumor types, given its role in many signaling pathways [141]. Inhibiting *DYRK1A* may have therapeutic potential in neurodegenerative disease models.

Several inhibitors of *DYRK1A* decrease tau phosphorylation and Aβ production, improving memory in 3xTg-AD mice [146]. In addition, KVN93, a *DYRK1A* inhibitor, has been shown to enhance long-term memory by promoting functional dendritic synapses in the 5xFAD model [147]. These studies suggest that *DYRK1A* may be a good therapeutic target. Although many inhibitors against *DYRK1A* have been identified, none have been brought into clinical trials. However, the development of more potent and selective inhibitors is required before human testing. The search for new types of inhibitors may provide new avenues for drug development and therapeutic intervention [141].

In this context, the methods in silico have already become indispensable tools, among them being molecular docking and ML. ML has been highly efficient in the identification of lead compounds from large databases. ML is currently at a rapid development pace, significantly enhancing the efficiency of compound identification and thus the speed of the early drug development phases [148]. Deep learning (DL), a subset of ML that employs artificial neural networks, often provides superior performance through its complex, hierarchical structure known as a deep neural network (DNN) [141,148]. In drug discovery and design, a DNN processes molecular features as inputs, performing calculations based on a specified set of parameters across multiple hidden layers. The model can be refined using back-propagation to achieve optimization. Integration of DL models in the process can potentially increase the speed of the identification of candidate compounds during screening by many folds [90].

### 6.2. Random Forest (RF)

Large ensemble machine learning algorithms, however, such as random forest (RF), have tended to perform better in neurological disease diagnoses compared to single classifiers. Random forest is a powerful ensemble machine learning technique widely used for both classification and regression tasks. It operates by constructing multiple decision trees with random variations: each tree is trained on a bootstrap sample of the data, and at each node, a random subset of variables is selected to determine the best split. Predictions are aggregated across all trees using majority voting for classification tasks or averaging for regression tasks. This methodology offers several advantages, including reduced risk of overfitting, improved stability in high-dimensional datasets, and the ability to assign importance scores to features, making RF particularly well suited for complex datasets such as those in microbiome and neuroimaging research [91,149,150]. The additional feature of RF is its intrinsic feature selection that assigns an importance score to features, reducing the variable space [151].

In microbiome research, RF excels at classifying microbial communities and predicting health outcomes, including mental health conditions, based on gut microbiota composition. Its ability to handle high-dimensional data and estimate feature importance helps identify specific microbial species linked to various health conditions [91]. Similarly, in neurological disease diagnoses, RF has consistently outperformed single classifiers due to its robustness in handling outliers and its capacity for intrinsic feature selection [149,152].

Random forest has been extensively applied in AD research, particularly using structural MRI data. Studies based on the Alzheimer’s Disease Neuroimaging Initiative (ADNI) demonstrated that RF models achieve high classification accuracies, such as 90.3% for distinguishing AD from healthy controls (HCs) and 81.3% for differentiating MCI from HCs [153,154]. The challenge of distinguishing MCI from either AD or HC lies in the overlapping clinical and neurodegenerative features of MCI, which make it an intermediate stage between normal aging and AD [155]. Despite this, newer RF models have shown improved accuracy, especially when incorporating a comprehensive set of features, including cortical thickness, subcortical volumes, and cognitive data [149,156]. For example, an RF model leveraging cortical and subcortical volume metrics demonstrated superior performance compared to conventional tools like Freesurfer, achieving 93.5% accuracy for distinguishing AD from HC, with an area under the curve (AUC) of 0.99, versus Freesurfer’s 91.9% accuracy and 0.98 AUC. In MCI classification, the RF model also performed comparably to prior models, with 80.8% accuracy in distinguishing MCI from HC or AD [156]. However, the general performance of RF models is still better for AD versus HC classification compared to the more challenging MCI-related classifications [86,157].

Feature importance analysis in RF models has provided valuable insights into disease pathology. For distinguishing AD from HC, critical regions such as the hippocampus, inferior lateral ventricle, and amygdala align with established patterns of cortical atrophy in AD. Interestingly, regions like the fusiform gyrus, cerebral white matter, and insular cortex, which emerged as significant in some RF models, suggest novel areas for investigation and highlight the potential of RF to uncover new biomarkers. These findings underscore the need for further studies with larger datasets to validate the contributions of these regions [158].

Despite its strengths, the application of RF in clinical practice faces limitations. Many studies rely on structural MRI datasets collected using older 1.5T scanners, while modern 3T MRI systems offer greater diagnostic reliability. Additionally, preprocessing techniques like Freesurfer, commonly used for brain segmentation, are time-consuming and lack reproducibility across methods, posing challenges to scalability in clinical settings [149,158,159,160]. There is a growing need for quantifiable indices reflecting continuous structural changes in the brain, such as cortical volume and thickness, to better capture the progression of neurodegeneration in AD. Technical advancements, including faster and more reproducible segmentation methods, are essential to expanding the clinical utility of RF-based diagnostic systems [161].

Random forest continues to play a vital role in advancing AD research by offering a robust, accurate, and interpretable approach to classification. Its integration with diverse feature sets, including imaging and cognitive data, holds promise for improving diagnostic precision and providing new insights into the disease’s progression [110,162]. Future efforts should prioritize larger, more diverse datasets and further technical refinements to enhance RF’s feasibility in routine clinical applications [149].

### 6.3. K-Means Clustering

J. A. Hartigan and M. A. Wong proposed the clustering idea in 1979 [163]. Since then, the technique has been significantly improved and nowadays is a very popular, predictive machine learning approach to uncovering a hidden relationship between feature sets and their grouping. The ease of implementation, together with the powerful categorization capabilities, makes K-means clustering particularly appreciated within a wide variety of applications in medical and diagnostic fields [164]. This unsupervised learning technique involves the high-speed division of data into clusters according to the similarity of features [165]. Microbiome profiles are groupings of individuals with similar mental health conditions, bringing out patterns among them. The algorithm is simple and efficient, particularly appropriate for exploring large datasets to identify patterns or clusters in complex microbiome data [166,167].

### 6.4. Support Vector Machines (SVMs)

Support vector machines (SVMs) are a supervised algorithm, appropriate for classification and regression tasks. SVMs classify accurately by finding an optimal hyperplane that separates the classes of given data. These ML algorithms, like k-means clustering and support vector machines, are used in the classification and grouping of data coming from microbiome studies, which allows researchers to classify different types of gut bacteria and find patterns related to mental health conditions and their predictions [168]. Such classification is made by classifying bacteria types associated with different mental health states based on microbiome profiles. Some of the positive features of SVM include handling high-dimensional data, working very well with small-to-medium-sized datasets, and being flexible for different applications [169]. Additionally, by utilizing wireless inertial sensors to measure head, pitch, roll, and stride rotations and analyzing the data, SVM can accurately classify PD patients and healthy individuals with over 90% accuracy [170].

### 6.5. Naive Bayes (NB)

Naive Bayes is a widely used probabilistic algorithm in text classification and spam filtering that assumes feature independence. Random forest and decision trees (DTs) are ensemble methods; RF combines multiple decision trees to enhance accuracy and robustness, while DT partitions data into subsets based on features to make decisions. Linear Discriminant Analysis (LDA) and Quadratic Discriminant Analysis (QDA) are applied to classification tasks, with LDA identifying linear decision boundaries and QDA detecting quadratic ones [171]. In the study by Miltiadous et al., both decision trees and random forests were tested, and the DT (C4.5) showed outstanding accuracy [172]. Various machine learning algorithms were compared using Fast Fourier Transform (FFT) and Continuous Wavelet Transform (CWT) features, with K-Nearest Neighbors (KNN) yielding the best classification accuracy in all cases [173]. The combination of CWT and Bicoherence Spectrum (BiS) features improved classification performance, with the multi-layer perceptron classifier outperforming other models [92]. Ruiz-Gómez et al. explored multiclass classification methods (LDA, QDA, and MLP) to classify data by trials and subjects [93]. Complexity-based features, such as Spectral Entropy and Zero Crossing Rate, were classified using K-nearest neighbor, as explored by Kulkarni et al. [94]. Additionally, various classifiers such as MLP, KNN, support vector machines, naive Bayes, and decision trees were evaluated for whole-brain dynamics [174]. Studies by Vecchio et al. and Nobukawa et al. employed SVM classifiers for their research [175,176].

A study proposed the DCCA cross-correlation coefficient to measure cross-correlation between EEG electrodes in AD patients [177]. In another study, K-NN outperformed SVM and DT classifiers as the best classification algorithm [38]. Cicalese et al. performed feature selection using the Pearson correlation coefficient and LDA for classification based on EEG and fNIRS-derived features [178]. Song et al. applied KNN, NB, and CART decision tree methods for classification based on specific brain connections [179]. These machine learning algorithms reliably predict early detection of Alzheimer’s using the broad availability of datasets for decision-making. The combination of CWT and BiS features improved classification, with the best performance achieved using multi-layer perceptron (MLP) [92]. The multi-layer perceptron used in studies by Lee et al. and Albright et al. is a feedforward neural network with multiple layers, well utilized in deep learning for complex tasks such as image recognition [180,181].

### 6.6. Deep Learning (DL) and Pattern Recognition

Deep learning is a domain of artificial intelligence that deals with generating advanced models inspired by the human brain’s neural networks. It employs complicated architectures built from several layers of nodes/neurons interconnected, forming a neural network, each of whose layers extracts a feature from the input data. Starting layers capture basic features, while those deeper build on them with the ability to recognize more sophisticated patterns. As a branch of ML, it uses multi-layered neural networks to analyze complex datasets, transforming microbiome research [182]. Among the most significant applications of DL is pattern recognition. Techniques like CNNs and recurrent neural networks (RNNs) can be extremely powerful in detecting subtle patterns within microbiome data, which may go unnoticed by standard analysis. These neural networks thus contribute to identifying weak connections between gut bacteria and brain function or behavior, helping to better understand the microbiome–GBA [83,183].

Deep-learning architectures, including CNNs for image processing and RNNs for sequential data, are especially capable of learning representations from raw data [184,185,186,187]. These deep learning models are effective in processing data with grid-like structures, such as images or spatial data. In microbiome studies, CNNs pinpoint spatial relationships and visual patterns in microbiome composition data that capture local and hierarchical structures in a high-resolution dataset. CNNs automatically learn patterns in data without any manual input; thus, they will become extremely powerful in microbiome research [188]. Unlike traditional feedforward networks, RNNs have directed cycles, which make them retain and make use of information from the past. This capability of memory makes RNNs very suitable for tasks that include sequential data, such as time-series microbiome analysis, whereby they can trace temporal changes in microbial compositions and investigate their sequential relationship with mental health states. Their capability of modeling complex temporal dependencies is what makes RNNs valuable in longitudinal microbiome studies [189].

**Figure 3:**
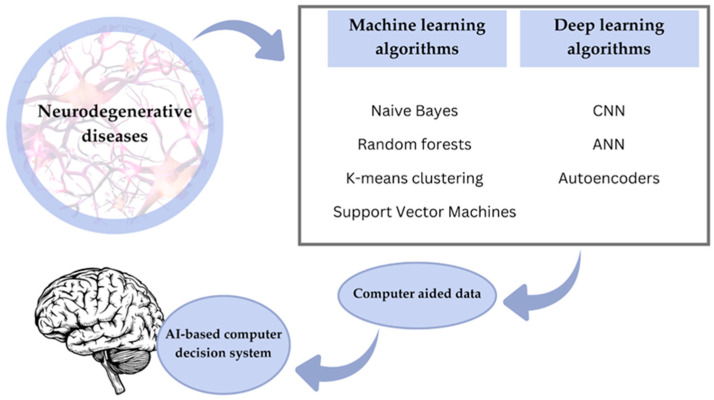
AI-based machine learning and deep learning in neurodegenerative disease diagnosis. The figure outlines the application of AI in the diagnosis of neurodegenerative diseases, including Alzheimer’s and Parkinson’s diseases. It draws on a range of machine learning algorithms, such as naive Bayes, random forests, K-means clustering, and support vector machines, as well as deep learning approaches like convolutional neural networks (CNNs), artificial neural networks (ANNs), and autoencoders. These are techniques that handle complex data to enhance diagnosis and features. The flow represents the role of computer-aided data in supporting AI-based decision systems by representing how structured data feeds into algorithms to improve clinical outcomes.

These models improve through iterative processes of forward and backward propagation, which optimize the parameters to minimize the gap between the predictions and actual results. This enables them to make accurate predictions and handle complex tasks. Generally, convolutional neural networks, which were initially used for MRI and PET scans, are considered very prominent for AD detection. These deep learning architectures are critical in early detection by performing different imaging modalities for Alzheimer’s dementia using various datasets. The first few layers in these models detect low-level features, edges, or textures, and further layers combine these low-level features to detect complex patterns linked with AD. The depth and computational power of deep learning let subtle biomarkers and abnormalities, often the harbinger of the disease, be detected. This capability for early detection offers significant potential for prompt intervention and better management of AD [39]. Figure 3 summarizes the most important aspects of AI-based machine learning and deep learning in neurodegenerative disease diagnosis.

## 7. Biochips as a New Trend

**Figure 4:**
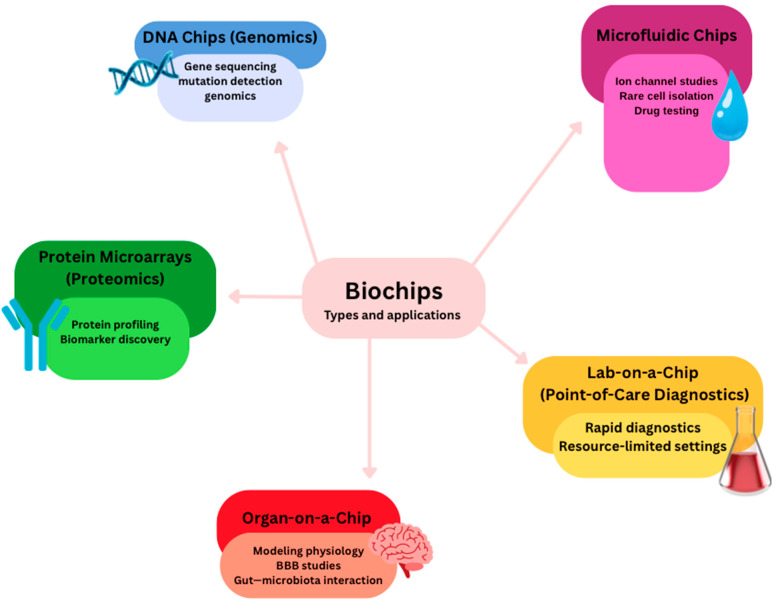
Types and biomedical applications of biochips: diagnostic, therapeutic, and research tools in molecular medicine. The figure illustrates the major categories of biochips and their applications across different domains of molecular medicine. DNA chips (genomics) are employed for gene sequencing, mutation detection, and genomic analyses. Protein microarrays (proteomics) enable protein profiling and biomarker discovery. Microfluidic chips are utilized in ion channel studies, rare cell isolation, and drug testing. Lab-on-a-chip devices (point-of-care diagnostics) facilitate rapid diagnostics, particularly in resource-limited settings. Organ-on-a-chip systems allow physiological modeling, studies of the blood–brain barrier (BBB), and investigation of gut–microbiota interactions.

There are many types of biochips, used for a variety of purposes (Figure 4). Together, these biochip technologies provide versatile platforms for diagnostics, therapeutics, and translational research in molecular medicine. Biochip technology integrates the fields of semiconductor microfabrication, nanomaterials, and biochemistry to develop small devices for biomedical purposes [190]. In the sense of biosensing, a biosensor couples a biological recognition element, like enzymes or antibodies, through a transducer for identifying and quantifying analytes utilizing detectable electrical signals [191,192]. Miniature biochips find broad applications in the high-throughput detection of pathogens, biomarkers for diseases, and environmental pollutants [193,194,195,196]. Applications include DNA chips in genomics, protein microarrays in proteomics, and microfluidic platforms in ion channel studies. Further, single-cell microfluidic platforms are capable of rare cell isolation; lab-on-a-chip systems allow for quick diagnostics, whereas point-of-care testing enables detection in resource-limited areas [194,197,198].

### 7.1. Optically Transparent Microfluidic Culture-Chips

Optically transparent microfluidic culture-chips allow for monitoring in real time, optically, the encapsulated or separated living cells, spheroids, or microorganisms and, thus, are very important for carrying out studies on biological processes in a controlled environment [193,198,199]. Several different microfluidic designs, such as gradient generators for chemotaxis, hanging-droplet generators for 3D spheroid cultures, hydrodynamic trapping systems, and oil–water droplet generators for single-cell heterogeneity studies, extend their functional capabilities. Among these, the organ-on-a-chip (OOC) platforms stand out. The latter is made up of microfluidic channels, wells, or even hanging droplets that include the living cells. All these integrate to actively manipulate physiological conditions for the facilitation of the modeling of diseases and testing of drugs [199,200,201].

OOCs employ controlled fluid flow, mechanical forces, and biochemical gradients that allow cells to develop into miniature models of tissues, including, but not limited to, the liver, lung, heart, kidney, brain, and intestine. This ability allows researchers to study organ-specific processes and diseases [200,202]. In contrast, OOCs offer some advantages over traditional 2D cultures by offering a 3D cell-culture environment that is very similar to physiological conditions [203]. This is essential for the understanding of complex drug interactions and can be realized using multi-channel high-throughput testing for extensive data gathering. Besides this, OOCs are an ethical way to test alternatives to animals by reducing the number and time taken for doing experiments in drug development, hence reducing the costs. Yet, because of their complexities, they cannot completely replace animal models [190].

One of the major applications is the modeling of the BBB a selective interface between neural tissue and blood, crucial for neuropharmaceutical delivery in pathologies such as Alzheimer’s disease. Initial efforts utilized static brain endothelial cell culture in Transwell plates and the investigation of other BBB components, including astrocytes and pericytes. Newly developed bio-microfluidic chips can achieve more advanced control over microscale architecture and physicochemical signals, enabling the overcoming of significant limitations of the traditional models and enhancing the relevance to human BBB studies [200].

The second key application involves gut microbiota, which plays a profoundly important role in digestion, nutrient absorption, neurotransmitter synthesis, and immunoregulation. Intestinal cells and gut microbiota from human fecal samples are cultured on gut-on-a-chip (GOC) platforms to study host–gut microbiota interaction and test different therapeutics. In one such attempt, Yuan et al. introduced a device fabricated by layering PDMS, separated with a polytetrafluoroethylene membrane [204]. They have used this to mimic the blood–gut barrier in the study of the interaction between probiotics and bacterial biofilm with epithelial cells. GOCs, in turn, enhance our knowledge about microbiome–host–immune interaction by accurately mimicking the environment inside the gut [190].

It is expected that AI algorithms will be able to simulate the optimal microfluidic design of OOCs and analyze such complex data generated by these systems. AI will assist in pattern identifications, insight extraction, and fast-tracking drug testing and disease modeling to gain a better understanding of the biological mechanisms of diseases related to the GBA [205,206].

### 7.2. Biochips for Neurostimulation

Brain–omputer interfaces (BCIs) are systems that form a direct communication between the brain and various external devices by detecting brain signals, such as EEG signals, and translating these signals into commands to control these various external devices, bypassing motor pathways [190]. There are two common methods: non-invasive approaches using scalp electrodes and invasive approaches using intracranial recordings with cortical or deep-brain electrodes [207]. However, these can detect deeper activities of the brain [190]. Where EEG signals are quite weak and prone to interference, more reliable data can be provided by intracranial EEG and neural implants.

Neural implants, including DBS devices, bioelectronic actuators, and neurostimulation chips, are small-sized biomedical devices with the ability to sense and provide electrical pulses for therapeutic purposes [208,209,210]. Brain-chip technology is both therapeutic and augmentative. Applications range from restoring sensory and motor functions, controlling external devices, and enhancing cognitive capabilities [207,211].

Recent developments in conductive hydrogels have improved the reliability and tissue bonding of implantable or wearable BCIs and biochips [212,213]. These hydrogels can present ionic and electronic conductivity, where ionic conduction requires free ions that are usually introduced by electrolytes, while electronic conductivity can be enabled by the introduction of metal nanoparticles, graphene, or conductive polymers [214,215]. However, the microscale biochip attachment to tissues due to weak van der Waals interactions is hard and unstable on dynamic tissues, also [216]. Regarding that, Kondaveeti et al. proposed a conductive hydrogel by using polyacrylic acid with other components showing stretchable and self-adhesive features. New developments in flexible and implantable electronics have improved neuromuscular system stimulation and recording and have made neural implants more user-friendly with higher-quality signals [211,215,217,218].

However, they are still associated with several risks and ethical concerns regarding long-term biocompatibility and invasive surgery. Schiff’s method measures the information rate of a BCI system implanted inside a patient’s brain. It uses such a measurement as a feedback variable to adjust stimulation for some ideal information passage [219]. Ortiz et al. have also developed a BCI that decodes gamma band activity and attention levels during the performance of motor imagery tasks. These help in mobility for persons with paraplegia or tetraplegia and are useful in rehabilitation following neurological conditions [220].

### 7.3. Utility of Biochips

Major advances in bio-sensing technology have taken place in the 21st century due to improvements in microfabrication and miniaturization of devices. These advances facilitate the integration of multiple smart and small-scale systems for the development of better bio-sensing through sample processing, fast detection, and instantaneous feedback using wireless communication. Lab-on-a-chip platforms serve biomedicine in terms of point-of-care testing and high-throughput analyses [194,221]. However, challenges remain prominent at the level of data complexity, which prevents effective interpretation by practitioners.

Real-time processing of data acquired from continuous monitoring of vital signs and biomarkers is indeed crucial for supporting the diagnosis of diseases in clinical settings. AI algorithms can efficiently analyze large data generated by biochips for patterns and anomalies that may signify diseases like cancer and infectious diseases and ensure microbial safety [193,194,199,221,222]. Integrated biochips with AI/ML shall enable real-time monitoring and analysis of biological processes, thus offering personalized medicine for improved patient outcomes. Moreover, AI/ML-integrated lab-on-a-chip platforms can shift their dependence on skilled data analysts, making the use of such lab-on-a-chip devices feasible even for resource-poor environments.

Biochips can possess lots of advantages, referring to disease modeling and testing of new drugs by diminishing animal use and accelerating the testing processes, specifically OOC systems. AI and ML algorithms can cope with processing and analyzing complex data problems for enhancing automation, pattern recognition, and outcome prediction in an attempt to further improve the effectiveness of applications in medicine. In addition, deep learning has provided outstanding performance for MRI, ultrasound, and computed tomography medical imaging applications [223,224]. It enables quality video-rate imaging, high-throughput image acquisition from biochip chambers, and fast image analysis to support predictive medicine [225]. However, scarce clinical sample availability is a challenge for training models. The application of AI-assisted imaging chips in the future will, therefore, do robust data processing and provide analytical precision, making great contributions to early disease detection, personalization of patient profiling, and the development of tailored therapies [207,226].

### 7.4. BIOCARD Data

Studies from 2022 also suggest that AI approaches can predict the cognitive trajectory of healthy individuals, which could permit a pre-dementia stage. For example, Przybyszewski et al. used granular computing rules to analyze cognitive data from the Biomarkers of Cognitive Decline Among Normal Individuals (BIOCARD) study that tracked 354 healthy participants for over two decades. In this study, the results show that AI methods may spot cognitive signals of early dementia in normal subjects not apparent to neuropsychologists at all [227]. For more than twenty years, the cognitive status of the participants was reviewed yearly as normal, MCI, or dementia. This study used the Clinical Dementia Rating Sum of Boxes (CDRSUM) as a quantification for assessing mild dementia and, based on it, developed a rough set of rules of classification. Finally, CDRSUM scores were used to classify the participants as having AD, or MCI, or being cognitively normal. They found that some of them presented with undiagnosed possible cognitive impairment or mild dementia by neuropsychologists. These findings emphasize AI’s capability of detecting even slight changes in cognitive abilities, which can indicate pre-dementia conditions [228]. This research and these discoveries are an important step toward the diagnosis of AD at an early stage. The patterns in the cognitive features of normal subjects can show, through the use of AI, early signs of dementia; hence, there will be time for intervention before the beginning of clinical symptoms.

Another application using multi-granular computing sharpens the classification of cognitive data for early detection of AD within the framework of the BIOCARD study [229]. Through the BIOCARD study, researchers compared the number of attributes from a formerly fixed set of fourteen; hence, the granules expanded from five to seven attributes. This allowed the comparison of the classification outcomes in a more detailed manner. Emphasis was also put on the interpretability of the rules derived from various granular levels. The authors sought to develop complete and consistent classifications across diverse rule sets by generating rules with different granularities and algorithms. All these will hopefully contribute to establishing an early diagnosis system that is more precise and robust, hence highly essential for effective intervention. Researchers have various models in testing with the view of ascertaining which best indicates the difference in stages of diseases like Alzheimer’s and Parkinson’s diseases. This can be done while emphasizing the classification that will not change due to the algorithms used or otherwise. The ultimate aim is to reach an even more precise diagnostic tool for timely intervention in diagnostics [230].

## 8. Organ-on-a-Chip

Research on the involvement of microbiota in neuropathy has been difficult, mostly because an in vitro model of the microbiota–GBA is lacking. This has significantly restricted the understanding of how microbiota might interact with the nervous system. In this respect, bioengineering approaches focus on advanced technical equipment and 3D engineering models to improve the dependability of in vitro tools. The “organ-on-a-chip” microfluidic system represents another novel approach in the bioengineering research field in which lab-on-a-chip technology is combined with 3D organ cultures [231,232,233,234]. These miniature-sized OOC devices have the potential to work as micro-bioreactors and, through continuous perfusion of culture media, maintain long-term in vitro culturing. They may reproduce relevant features of physiological and pathological conditions of tissues and organs in vivo in the absence of animal models; therefore, they may contribute to reducing costs and increasing efficiency while minimizing ethical problems related to the use of animal models for testing therapeutic agents [235].

So far, different tissues and other body structures have been modeled using different organs-on-a-chip. Examples include mimicking the transport of molecules across microanatomical barriers, such as the BBB, and even culturing intestinal microbiota. This allows for the study of interactions between the microbiota and other organs. An example is the coculture of *Bifidobacterium bifidum* with intestinal epithelial cells in a dual-channel intestinal microarray for the study of the bacterium in inflammatory bowel disease [236]. Intestinal disease symptoms were induced initially within the microarray devices using TNF-α and LPS. With this model, sharp gradients across the intestinal epithelium can be set up by modifying microchannel dimensions, occluding oxygen permeation through specific portions of the PDMS layer, and controlling hypoxic/aerobic medium flow. The results revealed that *Bifidobacterium bifidum* stabilizes the intestinal epithelial barrier by suppressing perturbations and promoting active recovery of injured cell monolayers [235].

Linking these systems together is a considerable technical challenge in modeling across body systems and microanatomical barriers to the transport of microbiota-derived neurotoxins, which is required for the investigation of the hypothesis behind the microbiota–brain axis. This necessarily makes transitioning from a microbiota–brain microarray approach to a more complex “multi-organ microarray” model a promising alternative to animal testing.

### 8.1. Gut-on-a-Chip

Gut microarray systems represent a robust in vitro system for the study of physiology, pathology, and pharmacology in the human gut. Gut microarray systems have huge potential to understand and treat a wide array of intestinal diseases like inflammatory bowel disease (IBD) and colorectal cancers. Moreover, gut microarrays may further extend personalized medicine and drug screening technologies. The contributions of these systems to the study of pathophysiology and drug development, coupled with recent advances in gut-on-a-chip technology.

Gut microarrays developed to date have been intended for the study of fundamental gut functions and responses to environmental factors, drugs, and other forms of stimulation. For example, Shin et al. fabricated an in vitro intestinal model by integrating microfluidic microarrays with Transwell systems and successfully produced intestinal morphogenesis [231]. The model herein presents the fabrication of a gut-on-a-chip microfluidic device and hybrid chip with a Transwell insert for intestinal epithelial cell culture on either a porous polydimethylsiloxane (PDMS) or polyester membrane, hence inducing 3D morphogenesis. This method makes use of physiologically relevant shear stresses and mechanical movements to recapitulate the in vivo context without resorting to complex cell engineering. One other study fabricated a two-channel chip with an oxygen gradient that was controlled to study the therapeutic effect of *Bifidobacterium bifidum* on IBD [234]. In this model, the dimensions of microchannels were manipulated and oxygen permeation in the PDMS layer was blocked selectively to create the oxygen gradient required to satisfy the requirements of both the aerobic epithelial cells (CaCo-2) and the anaerobic bifidobacteria (*B. bifidum bifidum*). Therefore, colonization of the chip with *Bifidobacterium* increased the stability of the intestinal epithelial barrier (IEB) and was a first step toward the multifactorial modeling of IBD that can be used in the study of disease mechanisms, drug testing, and personalized medicine.

Further, this gut-on-a-chip model can also be applied to study gut–immune interactions. Recently, an immunoreactive microbiota–gut–gut axis chip platform mimicked the intestinal microenvironment concerning microflora structure and vertical morphology [236]. This model established a physiological oxygen gradient, therefore, from the serosal membrane to the intestinal lumen, satisfying conditions for the development of immunomodulating mediators and a bioactive extracellular matrix (ECM). Accordingly, this setup pointed out the anti-inflammatory role of probiotics as *Bifidobacterium longum* proliferation was stimulated by LPS-induced inflammation. The study also unraveled the role of microbiota in ECM remodeling, collagen alignment, and improvement of gastrointestinal immunity, as evidenced by immune mediators such as ROS and cytokine post-inflammation.

The construction of multi-organ models is central to in vitro systems in modeling biological responses. For instance, an OoC model of gut–liver was developed for the study of intestinal permeability and metabolism and their interaction. This system, using CaCo-2 and HT29 cells in the intestinal compartment with primary hepatocytes in the liver compartment, allowed for the evaluation of mycophenolate mofetil metabolism [233]. The formation of active mycophenolic acid to further glucuronide metabolites was evaluated as a function of time. Mechanistic modeling predicted drug clearance and permeability, incorporating gut–liver OoC data within silico models to interrogate complex intestinal and hepatic interactions. Taken altogether, the approach in this study—particularly parameter identifiability and sensitivity analysis—supports the extension of such methods to multi-organ models in the study of drug metabolism and toward an improved understanding of biological effects.

### 8.2. Brain-on-a-Chip

Having been one of the most complicated organs in the human body, so far, the brain has been able to be simulated in vitro through the construction of a bionic brain organoid. This has provided a powerful promise toward personalized medicine [237]. Neurodegenerative diseases (NDDs) have higher rates these days compared to recent decades; therefore, there has been a huge interest in developing new biological platforms for the study of diseases and testing of drug efficacy. The BBB, one OoC model with the capability to truly recapitulate barrier function within the brain, provides unparalleled opportunities in the study of the mechanisms underlying NDDs.

Recently, an organ-on-a-chip technology model of a human brain chip was developed that is representative of the substantia nigra region to study disruption of the BBB in PD due to aggregation of αSyn [238]. In this organ-on-chip model, the vascular-neuronal interface is reconstituted and populated with human iPSC-derived brain endothelial cells, pericytes, astrocytes, microglia, and dopamine neurons. Further validation of this model will be done by simulating abnormal αSyn aggregation conditions, where the response of the brain chip to such abnormal aggregation will be gauged. This model is important in drug target identification and therapeutic efficacy evaluation in PD and other synucleinopathies. Another study developed a functional neurovascular unit on a microfluidic chip as a model of ischemic stroke [239]. The model enhanced the knowledge of blood–brain barrier function and therapeutic stem cell interaction with host cells, which include brain microvascular endothelial cells, pericytes, astrocytes, microglia, and neurons. With this system, investigators tracked the brain infiltration of stem cells and the expression levels of genes from post-stroke pathology; all types of stem cells were driven by endogenous repair rather than direct cell replacement [240]. The identified synaptic activity restoration depended on the structural and functional integrity of the neurovascular units, rather than neuronal regeneration. It, therefore, offers a dependable platform for screening different types of stem cells that are under clinical investigation for stroke, with a wide application potential in vascular disease studies [239].

While advanced brain organoids and other brain models increase in size, the designs for brain-on-a-chip devices themselves are being developed into macroscale devices. Accordingly, neurospheres cultured under flow conditions indeed showed more intricate and robust neuronal networks than those grown under static conditions due to enhanced nutrient and oxygen supply, transport of cytokines, and removal of metabolic waste resulting from the slow, diffusive dominant flow at the interstitial level [241,242]. A typical example is a microphysiological stroke model of human cells in a 3D environment that emulates in vivo conditions. It is a reliable platform for testing the neural repair potential of stem cell therapies by simulating interactions with neurovascular units [239]. This platform offers a system testbed for evaluating, in terms of their repair behaviors, a wide range of various stem cell types currently under clinical investigation for the treatment of stroke. Moreover, the authors of the research, Cho et al., showed that microfluidic devices of the brain extracellular matrix, in turn, help to support functional and structural maturation of human brain organoids [243].

Such challenges include very long prototyping times and the lack of standardized operating procedures in the process of developing organs-on-a-chip. Investigators are also pursuing 3D-printed in vitro models of the brain [244,245]. Three-dimensional printing allows the manufacturing of central nervous system (CNS) models out of different materials, including living cells, providing biologically active 3D constructs modeling the CNS along the z-axis [246,247]. Such custom-designed, 3D-printed models are capable of generating verifiable neural tissues that would ensure uniformity in clinical research and drug screening. In this respect, a combination of 3D printing and microfluidic organ chips may provide a new approach to the engineering of bionic brain chips that can mimic an in vivo physiological and pathological process for translational research.

In vitro generation of brain models is indispensable for the further elucidation of the pathology not only of traumatic brain injuries but also of degenerative diseases, tumors, inflammation, and infections of the brain [248,249,250]. While researchers continuously improve the technology of organoids, human brain organoids still substantially differ from the real human brain, and this constitutes one of the big scientific challenges. Currently, this level of structural and functional complexity of real brain tissue has not been achieved: great differences in size, absence of functional blood vessels, and lack of neuroimmune cells in-vitro-cultured brain-like organs [237]. The gaps listed above are the main issues being investigated by a very large number of scientific teams. In that respect, with the global scientific effort, one is optimistic that a breakthrough will soon be achieved that will produce brain organoids close to emulating the human brain.

### 8.3. Gut–Brain-on-a-Chip

For decades, there has been an appreciation for the active communication between the brain and gut regarding physiological homeostasis. The investigation has demonstrated that gut–brain signals regulate basic physiologic functions as well as play a critical role in the pathogenesis of complex neurologic disorders [251,252]. Additionally, investigators are exploring this axis’s contribution to human health using in vitro model systems.

The GBA microfluidic system is a novel in vitro model useful for the simulation of communication between the gut and central nervous system, with several advantages such as modularity and high throughput [253,254]. This model is a valuable tool for the study of diseases and mechanisms related to the GBA [253]. In 2017, the European Research Council (ERC) funded Microbial Influences on Neurodegenerative Diseases and their Role in the Ecosystem of the Brain (MINERVA), the first bionic platform used to investigate the role of microbiota in neurodegenerative diseases like Alzheimer’s [255,256]. In addition, Min-Hyeok Kim et al. have also developed an intestinal microbiome-on-chip model, a modular microfluidic chip co-culturing intestinal epithelial cells with brain endothelial cells. This chip uses microfluidic channels to model the intestinal epithelial barrier and BBB, and it displayed responses that were consistent with microbial byproduct-mediated interactions between gut epithelium and the BBB, thus allowing for the first time the investigation of gut–brain communication via labeled exosomes [254]. Consequently, the GBA microfluidic chip can be quite versatile in the study of extracellular vesicles as carriers of drugs in vivo and their role within GBA [257]. In one such study, a GBA microfluidic chip system was applied to human-induced pluripotent stem cells (iPSCs)—derived neurons to explore the impact of metabolites and extracellular vesicles (EVs), which stem from gut microbes, on neurodevelopment and neurodegeneration. The results indicated that metabolites and exosomes across the GBA could induce neural differentiation and promote the expression of proteins associated with synapses in neurons [258].

In parallel with the microfluidic systems, for the identification of molecules and signaling pathways that enable communication between the gut and brain, an experimental gut–brain system was developed in a Petri dish integrated with a microporous cell culture membrane [259]. It contains a separate compartment for a “mini gut” consisting of endothelial cells and another one for a “mini-brain” consisting of crayfish nerves. The fluid continuum between these compartments enables the transfer and tracking of signaling molecules across these compartments. This platform has pinpointed serotonin as an important signaling molecule in communicating between the gut and the brain. Sensors in the platform reveal that serotonin (5-hydroxytryptamine) is efficiently transported across the endothelial surface into the subjacent layer [235]. Using the crayfish model, this platform recapitulates the natural electrophysiological responses following animal studies. This will enable the in vitro real-time simultaneous monitoring of the signals between the gut and the brain tissues, without necessarily having to revert to invasive procedures in humans or animals. More recently, a human gut–liver–brain physiological simulation system was also developed by using Microphysiological Systems (MPS) by researchers [260]. In this in vitro platform, brain cells are connected with colon and liver tissue models, permitting the flow of immune cells and nutrients, including short-chain fatty acids (SCFAs), through the channels linking them. This established model of immunometabolic cross-talk at present represents an important step forward in simulating the human GBA within the context of in vitro neurodegeneration.

**Figure 5:**
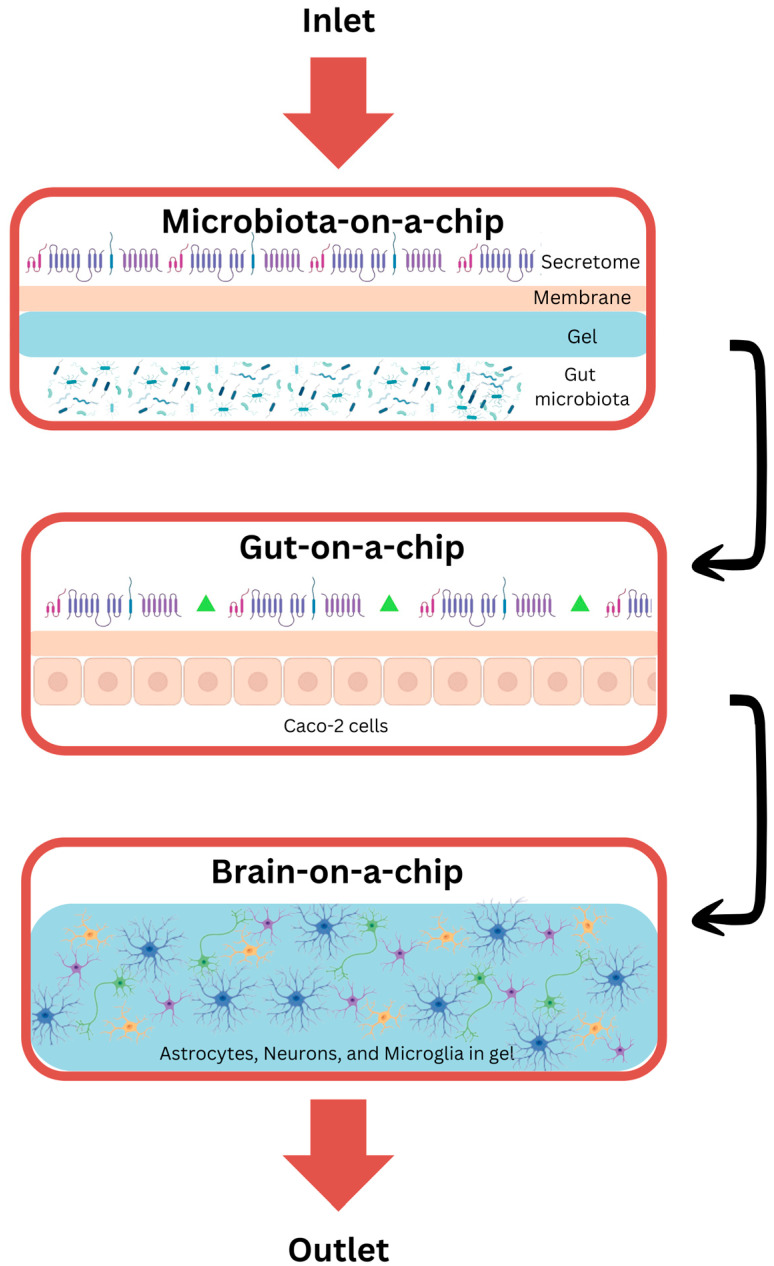
Integrated microbiota–gut–brain-on-a-chip system for neurological research. The integrated microfluidic platform is designed for the modeling of the microbiota–gut–brain axis. It is a system of three interconnected parts: the microbiota-on-a-chip, which models the gut microbiota environment by separating microbial secretions with a membrane and gel layers; the gut-on-a-chip, which models the intestinal epithelium using CaCo-2 cells to study gut barrier function and interactions with microbial metabolites; and the brain-on-a-chip, which models neural activity with astrocytes, neurons, and microglia embedded in gel to model the cellular microenvironment of the brain. These arrows show the bidirectional communications between the components, including an inlet through which to introduce the nutrients or stimuli and an outlet for output collection. This advanced platform will enable the study of the dynamic interactions within the microbiota–gut–brain axis for the investigation of translational research on neurological health and disease.

**Figure 6:**
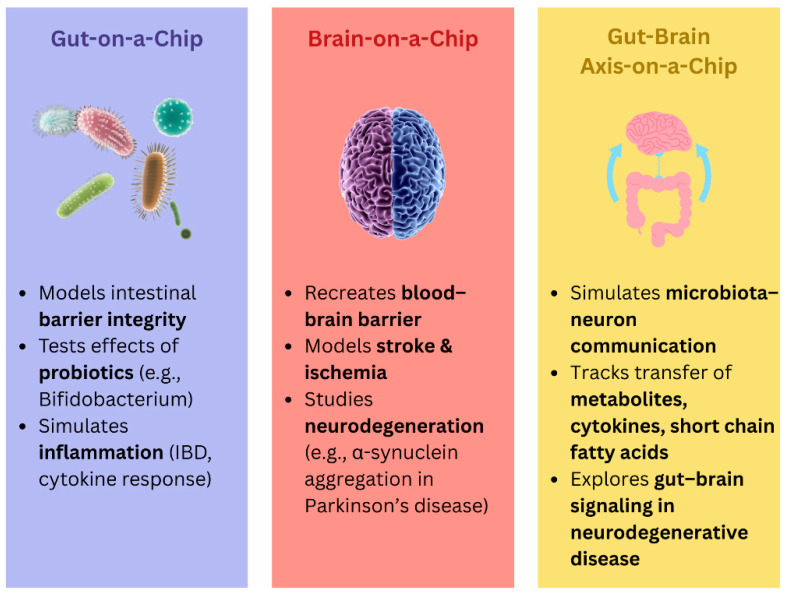
Comparison between gut-on-a-chip, brain-on-a-chip, and gut–brain-axis-on-a-chip.

Therefore, GBA chips present enormous potential concerning understanding the impact of the GBA on related diseases and provide a solid platform for therapy testing. Yet, several technological challenges exist in developing multi-organ microarrays, leading to only a few reported studies. Multi-organ chips, the GBA systems, have to obey some physical constraints regarding organ size, flow rates for each module, and total medium volume to maintain physiological relevance. Thus, this places a greater demand on chip design and fabrication [261]. It also remains a technical challenge to accurately recreate the physical and chemical microenvironments of in vivo tissues and organ dynamics in GBA chips [262]. Also, due to their complexity compared to single-organ chips, GBA chips often face greater problems in fabrication and higher costs [263]. Figure 5 illustrates an integrated microbiota–gut–brain-on-a-chip system for neurological research, while Figure 6 emphasizes differences between gut-on-a-chip, brain-on-a-chip, and gut–brain-axis-on-a-chip.

## 9. Exposome

The exposome, or the totality of environmental exposures, is increasingly at the forefront of research on Alzheimer’s Disease/Alzheimer’s Disease-Related dementia (AD/ADRD). Despite that, little is known regarding how interventions targeting the exposome through either individual behavioral changes or policy-level measures may influence the AD/ADRD disease burden at the population level in real-world conditions and their cost-effectiveness. Agent-based modeling (ABM) is a computational approach that simulates population-level phenomena based on simple rules governing individual behaviors and interactions [264,265]. It is particularly useful for extrapolating macro-level implications from micro-level assumptions [266]. ABM has been used in bioinformatics to analyze biological interactions, understand complex processes, and predict behaviors under various conditions [267]. Its ability to incorporate individual heterogeneity—where agents possess unique traits influencing behaviors and outcomes—is a key strength, particularly valuable in personalized medicine [268]. This contrasts with system dynamics (SD) modeling, which focuses on aggregate patterns [269]. ABMs also serve as virtual laboratories, simulating interventions at both individual and population levels, offering insights into intervention timing, targeting, duration, cost-effectiveness, and potential unintended consequences [270,271,272].

Recent studies have explored ABM’s potential in exposome modeling, leveraging individual heterogeneity and embedding agents in virtual environments, from simple grids to detailed GIS-based systems. Models such as the SpatioTemporal Human Activity Model (STHAM) and the Agent-Based Model of Human Activity Patterns (ABMHAP) have generated realistic human activity patterns validated against survey and traffic data [273,274,275]. Chapizanis et al. developed an ABM incorporating population, time-use, road network, and air quality data to simulate urban Thessaloniki, identifying subgroups with the highest PM2.5 exposure and highlighting variability even among adjacent residents [276]. Similarly, an abstract of the environmental model was used to simulate PM2.5 exposure patterns based on personal monitor data. These models extend traditional ABM applications from pathogen exposure in healthcare and bioterrorism scenarios to broader environmental exposures, such as air and water contaminants [268,277]. Other ABMs have explored AD/ADRD development, including microbial initiation via the olfactory system, cellular pathways of neurodegeneration, and the impact of blood pressure management on AD/ADRD prevention [278,279,280]. These studies suggest ABM’s potential to identify key exposures and processes in AD/ADRD development, though no current models fully connect the exposome with AD/ADRD progression.

If ABM is to realize its full potential for modeling exposome effects on AD/ADRD, then models need to capture dynamic, lifelong changes in exposures influenced by social interactions, regulatory changes, and industrial practices. ABMs can model behavioral adaptations to environmental changes, social norms, and accumulated experience [281]. Sensitivity analyses can help address uncertainties, but robust longitudinal data are needed for model calibration and validation, especially in policy applications [270,281]. The flexibility of ABM also allows modeling complex nonlinear dynamics, cumulative exposures, and multi-level interactions, including gene–environment interaction. However, increased complexity requires careful consideration of essential system components [282]. One of the key strengths of ABM in exposome and AD/ADRD research is to allow examination of intervention strategies across both the individual and policy levels. For instance, ABMs have simulated responses of industry to water management policies, air cap-and-trade programs, and urban residents’ response to PM2.5 reduction policies [283,284]. These models have illustrated how environmental context and individual-level characteristics determine the varying nature of the intervention effect, thus indicating the necessity for capturing heterogeneity in models to make predictions more valid [283,285,286]. ABMs allow the modeling of a variety of interventions to estimate the population-level burden of AD/ADRD in terms of multiple individual factors and environmental contributors. Such models are normally based on synthetically generated populations using information from multiple data sources such as chemical exposure, neighborhood pollution, social and lifestyle behaviors, sociodemographic characteristics, co-morbidities, and genetic risk factors. For example, a synthetic population for the U.S. can be generated using nationally representative exposome biomonitoring and census data. Once calibrated and validated, these models enable testing different intervention scenarios to evaluate their impacts on the projected AD/ADRD burden, which is expected to grow from 7 million to 13 million individuals by 2050 [287].

Interventions can be considered both at the individual and the population levels. Individual-level interventions might include personal behavioral changes, such as dietary changes or a reduction in exposure to synthetic pollutants via home cooking and water filtration systems. Broader-scale population-level interventions could include federal standards for contamination of drinking water, such as proposed regulations for per- and polyfluoroalkyl substances (PFASs). These models also allow the estimation of the cost-effectiveness of various strategies by incorporating intervention costs alongside their potential economic benefits, which can help in informing resource allocation and policy development [268].

While ABM offers promise for modeling the impact of exposome interventions on population-level AD/ADRD, several challenges remain. Harmonizing diverse data sources—such as environmental exposures, individual behaviors, and intervention outcomes—across multiple time scales is a major hurdle [283]. Additionally, the best way to represent complex lifetime exposures, including gene–environment interactions, is still unclear [288]. Scalability to different locations also depends on access to detailed local data as exposome factors vary widely across regions and time [289].

To address these issues, new methods for measuring cumulative exposures are being developed, along with model architectures that capture human–environment interactions [281,290]. Large-scale projects, such as the Health and Environment-wide Associations based on Large Population Surveys (HEALS) initiative and Exposome of Aging: A Systems-based Approach to Understanding the Role of the Environment in Aging and Age-related Diseases (EXPANSE), aim to fill data gaps and will be valuable for future ABM research [291,292]. These efforts will complement causal modeling techniques like structural equation modeling and g-computation, strengthening evidence on the exposome’s health effects over the lifespan.

### 9.1. Future Directions of Exposome and AI

The complexity of the exposome, coupled with its constantly evolving nature, presents major challenges to understanding its role in neurodegenerative diseases like AD and AD-related dementias. A unified method of quantifying cumulative exposome burden over time is a necessary precursor to effective interventions. Precision environmental health proposes the use of “multi-omic burden scores” in tailoring interventions for subgroups with the highest exposure or vulnerability. However, this becomes challenging due to high-dimensional and sparse data, time-varying exposures across subpopulations, and shifting exposure profiles due to factors such as behavioral changes and regrettable substitutions in which harmful chemicals are substituted with equally or more toxic chemicals [293]. For example, single-pollutant interventions, such as the use of BPA-free products, may not lower overall exposure to plasticizers if replacement chemicals are equally harmful, thus diminishing any potential intervention benefit on risk for AD/ADRD [268].

Harmonized summary metrics are necessary when comparing results across studies. Liu et al. developed an Item Response Theory (IRT) approach to standardize exposome burden scores. This allows consistent metrics between studies with different datasets [290,294,295,296,297]. Such a strategy enables the integration of multiple features of exposomes, anchoring on the shared features. This gives a better representation than one-proxy monitoring can achieve, wherein there is a possibility of missing evolving patterns of exposure. Using these metrics, researchers can model various interventions, with special benefits accruing for high-risk genetic subgroups, to better elucidate gene–environment interactions that drive AD/ADRD development [268].

### 9.2. Artificial Intelligence = New Exposome Opportunities

These models commonly use supervised learning to make predictions on AD/ADRD risk using past data. However, newer self-supervised learning techniques are being developed that can identify intrinsic patterns without explicit labeling and training based on the contrasting or reconstruction of noisy data [298]. Consider Semi-Supervised Learning (SSL) revealing hidden relationships between the exposome and disease based on environmental, genomic, or behavioral datasets, including incomplete data. Applications of SSL in medical imaging, electronic health records, and RNA/DNA networks suggest its potential in exposome research, particularly when large datasets become available [299,300,301,302,303]. Generative AI models could predict high-risk areas for AD/ADRD or explain individual risk factors using attention-based techniques, providing deeper insights into how environmental exposures influence disease prevalence.

## 10. Future Research Directions: Bridging Microbiome and AI

The next-generation gut–brain axes will emerge from the intersection of microbiome research and AI, with likely treatments across neurology and mental health. This may also involve AI-driven modeling efforts to make sense of the complexity of microbiome data in neurological outcomes; hence, increasing our predictability, diagnosis, and treatment of gut–brain interaction disorders such as AD, PD, and depression.

Recent studies underline that the microbiome–gut–brain axis (MGBA) are one of the most important areas in which treatment models should be developed on an individual basis. For instance, AI is capable of analyzing large datasets of microbiome profiles to identify biomarkers predictive of neurodegenerative and neurological disorders. Machine learning models are under construction, which will be able to process EEG and microbiome data synchronously; this gives insight into patterns of cognitive impairment and brain activity associated with gut health [304]. Integration of microbiome and neuroimaging data with AI has the potential to revolutionize early diagnosis and management of neurodegenerative disorders, possibly including non-invasive disease progression monitoring [305].

AI can also help narrow down specific microbial strains that might be engineered to change the gut–brain communication pathway. This potentially helps in developing microbiota-targeted therapeutics for conditions such as schizophrenia, autism spectrum disorders, and anxiety disorders. The use of AI in MGBA analytics, though recently developing, does provide a diagnostic framework but perhaps most significantly opens up treatment options through diet, probiotic, and microbial vectors of therapy, which may modulate brain health in more selective ways [306].

**Figure 7:**
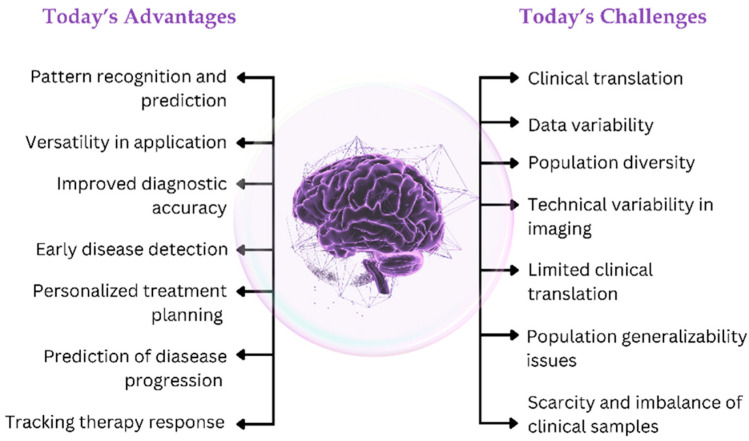
Advantages and challenges in AI applications for neurological research. This figure highlights the dual aspects of applying AI in neurology. Advantages include pattern recognition and prediction, application versatility, and improved diagnostic accuracy. However, challenges persist, such as clinical translation, variability in data, and population diversity, which limit the broader applicability of AI in neurological contexts. A central visual of the brain symbolizes the focus on advancing brain-related diagnostics and therapeutics.

In the sense of enhancing efficiency in medical and scientific research with the incorporation of AI, research procedures have gained much speed, while traditional research methods are generally time-consuming and limited. Similarly, AI will help automate the analysis of data and even generate hypotheses to increase the efficiency of research in the field of studying the MGBA at much faster rates. For instance, AI-driven analyses could rapidly point out the most promising research paths that could be omitted by researchers, thereby fast-tracking discovery [307,308]. AI is indispensable in analyzing large datasets, making predictions about outcomes, and identifying biomarkers and mechanisms at play in unraveling complex interactions between gut microbiota and the brain. The increased application of AI in the medical and scientific fields has expanded knowledge and opened new avenues of therapy in neurodegenerative diseases and early diagnostics. Figure 7 illustrates advantages and challenges in AI applications for neurological research.

## 11. Conclusions

The interplay of AI with neurodegenerative diseases, mainly AD and PD, has been discussed to improve our understanding, diagnosis, and treatment approaches. NDDS represent a high and rising global health burden characterized by complex etiologies, progressive impairments in cognition and motor function, and impacts on the quality of life of the patients. Hence, despite several decades of investigation, current approaches to diagnosis and treatment remain symptom-based, and urgent measures need to be undertaken to seek newer solutions.

AI has emerged as a revolutionary tool that holds great promise in unraveling the complexity of these disorders through large datasets, sophisticated algorithms, and machine-learning techniques. Its role in early detection, predictive modeling, and personalized treatment planning is also significant. In AD and PD, AI-driven neuroimaging and biomarker analysis allow for precisely identifying pathological changes, including amyloid plaques, tau tangles, and neural degeneration, long before the onset of clinical symptoms. These developments support earlier interventions that could alter the course of such diseases and improve patient outcomes.

One of the most exciting areas of research involves the GBA in which the role of the microbiome in neurological health is increasingly recognized. Dysbiosis or imbalance in gut microbiota has been implicated in the pathogenesis of neurodegenerative diseases. AI-powered tools have demonstrated unparalleled capabilities in analyzing multi-omics datasets, identifying biomarkers of dysbiosis, and modeling intricate interactions that involve the microbiome and brain health. Integrating genomic, metagenomic, and metabolomic data, AI provides a holistic view of the gut–brain interplay, hence opening new avenues for innovative therapeutic strategies targeting this axis.

Furthermore, AI enabled the development of a set of advanced neuroimaging techniques: segmentation, feature extraction, and predictive modeling. Such methodologies not only improve diagnostic accuracy but also allow for obtaining useful insights into disease progression and therapeutic efficacy. The role of AI in enhancing the quality of life in patients with PD, addressing both motor and non-motor symptoms, is further extended by AI-enabled applications, including robot-assisted gait training and brain–computer interfaces.

However, there is a set of challenges: challenges to be faced regarding data standardization and model interpretability, with ethical concerns in AI applications within neurodegenerative diseases research. For such studies to reach their full potential, clinicians, researchers, and technologists should jointly work in this area of AI applications in the responsible development of methods. The conclusion with a critical approach is as follows: AI can thus represent a paradigm change in fighting neurodegenerative diseases. Its ability to process vast and complex datasets, discover hidden patterns, and come up with personalized solutions offers unparalleled opportunities for advancing both our understanding and management of these conditions. AI can bridge the gap between the microbiome, neuroimaging, and clinical practice, holding promise for the transformation of neurodegenerative disease care into a more precise, predictive, and preventative discipline. While the research is continuously evolving, the synergy between AI and neuroscience is going to shape the future of healthcare, instilling hope among millions of people suffering from these debilitating disorders.

## Figures and Tables

**Table 1:** Molecular and systemic drivers of chronic neuroinflammation in Alzheimer’s disease and Parkinson’s disease.

Mechanism/Pathway	Key Mediators and Processes	Impact on Neurodegeneration	Source
Glial activation and cytokine release	Microglial and astrocytic activation; IL-1β, TNF-α, IL-6 secretion	Creates a toxic environment, synaptic dysfunction, neuronal death	[1,2,3,4,5,6]
Protein aggregates and DAMPs	Amyloid-β, α-synuclein, mitochondrial DNA, oxidized lipids	Engage PRRs (TLRs, NLRs); activate NLRP3 inflammasome; propagate inflammation	[6,21,22]
Inflammasome and autophagy dysfunction	NLRP3 inflammasome, cathepsin B, impaired lysosomal clearance	Self-sustaining inflammasome activation, reduced clearance of protein aggregates	[6,24]
HMGB1–RAGE/TLR4 signaling	HMGB1 redox states, ERK, MAPK, NF-κB pathways	Sustains pro-inflammatory phenotype, epigenetic remodeling of glia	[25,26]
Glutamate excitotoxicity	Reduced astrocytic EAAT1/2, excess extracellular glutamate	Postsynaptic calcium overload, neuronal apoptosis (hippocampus, substantia nigra)	[23]
Metabolic reprogramming	NF-κB-driven shift to glycolysis, lactate accumulation	Impaired mitochondrial biogenesis, extracellular acidification, myelin damage	[14,27]
Impaired resolution of inflammation	Reduced pro-resolving mediators (resolvins, protectins, maresins, lipoxins)	Failure to terminate inflammation, persistent microglial activation	[28,29]
Gut–brain axis and systemic inflammation	Gut dysbiosis, LPS translocation, TLR4–MD2–CD14 signaling, immunometabolism	BBB disruption, peripheral priming of microglia, chronic low-grade CNS inflammation	[9,10,11,12,13,14,15,16,17,18]
LPS-driven neurotoxicity	NF-κB, AP-1, IRF3, COX-2, iNOS activation; ROS production	Enhances tau/α-synuclein pathology, synaptic loss, mitochondrial dysfunction	[12,13,14,15,16,17]
Therapeutic innovations	Nanomedicine (BBB-penetrating nanoparticles, microbiota-targeting nanosystems); AI for data integration	Targeted modulation of inflammation, gut–brain homeostasis restoration, personalized therapy	[19,20]

**Table 2:** Computational approaches for Alzheimer’s disease.

Computational Approach	Main Use	Advantages	Disadvantages	References
Brain imaging with computer learning (MRI, PET, EEG)	Diagnosis (early detection, staging)	Captures structural, functional, and molecular brain changes; combines multimodal data; large datasets exist.	Accuracy varies across scanners and hospitals; requires diverse data.	[63,64,65]
Deep learning and transfer learning (neural networks on MRI/PET/EEG)	Diagnosis (detecting Alzheimer’s, predicting progression)	Learns complex features; pre-trained models improve accuracy; ensemble methods show high performance.	Needs large, annotated datasets; limited clinical translation so far.	[77,78,79,80]
Random forest models (on MRI and cognitive data)	Diagnosis (differentiating Alzheimer’s, mild cognitive impairment, healthy controls)	High accuracy (~90–93%); identifies key brain regions; robust to noise.	Less accurate for mild cognitive impairment; heavy preprocessing required.	[81,82,83,84]
Classic machine learning (support vector machines, gradient boosting, etc.)	Diagnosis (using MRI, EEG)	Works well with small/medium datasets; effective on EEG/imaging.	Dataset-dependent; single models may not generalize.	[53,85,86]
Unsupervised clustering (e.g., k-means)	Research and patient subgrouping	Simple; finds hidden patterns in MRI and microbiome data.	Exploratory only; requires validation.	[87,88,89]
Artificial intelligence applied to gut microbiome	Diagnosis and treatment (biomarkers, diet, probiotics)	Identifies microbial biomarkers; supports personalized therapies.	High variability; still early for clinical translation.	[90,91]
Artificial intelligence for drug discovery (in silico modeling, docking, machine learning)	Treatment (finding new medicines)	Accelerates screening; finds promising inhibitors.	Many targets not yet in clinical trials.	[92,93,94]

**Table 3:** Computational approaches for Parkinson’s disease.

Computational Approach	Main Use	Advantages	Disadvantages	References
Classic machine learning on wearable sensors (support vector machines, gradient boosting)	Diagnosis (movement monitoring, detection)	Achieves high accuracy (>90%) using movement sensors; wearable devices enable real-world use.	Dependent on dataset quality; clinical robustness still limited.	[95]
Artificial intelligence applied to gait training (robot-assisted rehab, deep learning on pressure data)	Treatment and monitoring	Robot-assisted gait training improves walking and endurance; deep learning detects freezing-of-gait in real time.	Optimal training programs unclear; long-term benefits uncertain.	[96,97,98]
Brain imaging with computer learning (MRI, PET, EEG)	Diagnosis (early detection, tracking disease progression)	Captures structural and functional brain changes; allows early detection.	Needs harmonization across centers; performance may drop on unseen datasets.	[53,95]
Artificial intelligence applied to gut microbiome	Diagnosis and treatment (biomarker discovery; guiding therapies)	Reveals associations between gut bacteria and Parkinson’s; supports personalized therapeutic strategies.	Data variability across studies; translation into clinical use still limited.	[90,91]

## Data Availability

No new data were created or analyzed in this study. Data sharing is not applicable to this article.

## Abbreviations

ABC	Artificial Bee Colony
ABM	Agent-Based Modeling
ABMHAP	Attention-Based Multi-Head Adaptive Pooling
AD	Alzheimer’s Disease
ADASYN	Adaptive Synthetic Sampling Approach for Imbalanced Learning
ADD	Alzheimer’s Disease Detection
ADNI	Alzheimer’s Disease Neuroimaging Initiative
ADRD	Alzheimer’s Disease and Related Dementias
AI	Artificial Intelligence
ALOX	Arachidonate Lipoxygenase
ALX	Lipoxin A4 Receptor
ANN	Artificial Neural Network
AP	Activator Protein
ATN	Amyloid/Tau/Neurodegeneration
ATP	Adenosine Triphosphate
AUC	Area Under the Curve
BAT	Bat Algorithm
BBB	Blood–Brain Barrier
BCI	Brain–Computer Interface
BIOCARD	Biomarkers of Cognitive Decline Among Normal Individuals
BPA	Bisphenol A
CART	Classification and Regression Tree
CDRSUM	Clinical Dementia Rating Sum of Boxes
CNN	Convolutional Neural Network
CNS	Central Nervous System
COX	Cyclooxygenase
CRP	C-Reactive Protein
CSF	Cerebrospinal Fluid
CWT	Continuous Wavelet Transform
DAMPs	Damage-Associated Molecular Patterns
DBS	Deep Brain Stimulation
DCCA	Detrended Cross-Correlation Analysis
DL	Deep Learning
DNA	Deoxyribonucleic Acid
DNN	Deep Neural Network
DT	Decision Tree
DYRK	Dual-specificity Tyrosine-Regulated Kinase
ECM	Extracellular Matrix
EEG	Electroencephalography
ERC	Extracellular Receptor Kinase (likely context-dependent, check text)
ERK	Extracellular Signal-Regulated Kinase
EXPANSE	Environmental Policy for the Ageing Population (project acronym)
FDG	Fluorodeoxyglucose
FFT	Fast Fourier Transform
FTLD	Frontotemporal Lobar Degeneration
GA	Genetic Algorithm
GBA	Gut–Brain Axis
GBM	Gradient Boosting Machine
GI	Gastrointestinal
GIS	Geographic Information System
GOC	Gut-on-a-Chip
HC	Healthy Control
HEALS	Health and Environment-wide Associations based on Large population Surveys (project acronym)
IBD	Inflammatory Bowel Disease
IEB	Intestinal Epithelial Barrier
IFN	Interferon
IL	Interleukin
IRT	Item Response Theory (context-dependent, check text)
KNN	K-Nearest Neighbors
LBP	Lipopolysaccharide-Binding Protein
LDA	Linear Discriminant Analysis
LOA	Lion Optimization Algorithm
LPS	Lipopolysaccharide
MAPK	Mitogen-Activated Protein Kinase
MCI	Mild Cognitive Impairment
MD	Mean Diffusivity
MGBA	Microbiota–Gut–Brain Axis
MINERVA	Mechanism-based Integrated Systems for Neurodegeneration and Disease Variation (project acronym)
ML	Machine Learning
MLP	Multi-Layer Perceptron
MMSE	Mini-Mental State Examination
MPS	Microphysiological Systems
MRI	Magnetic Resonance Imaging
MTAP	Multi-Task Attention Pooling
NADPH	Nicotinamide Adenine Dinucleotide Phosphate
NB	Naïve Bayes
NDD	Neurodegenerative Disease
NDDS	Novel Drug-Delivery Systems
NF	Nuclear Factor
NLP	Natural Language Processing
NN	Neural Network
NSD	Neuronal alpha-Synuclein Disease
OASIS	Open Access Series of Imaging Studies
OOC	Organ-on-a-Chip
PD	Parkinson’s Disease
PDMS	Polydimethylsiloxane
PET	Positron Emission Tomography
PFAS	Per- and Polyfluoroalkyl Substances
PSO	Particle Swarm Optimization
QDA	Quadratic Discriminant Analysis
RAGE	Receptor for Advanced Glycation End Products
RAGT	Robot-Assisted Gait Training
RF	Random Forest
RGB	Red, Green, Blue (color channels)
RNA	Ribonucleic Acid
RNS	Reactive Nitrogen Species
ROS	Reactive Oxygen Species
SCFA	Short-Chain Fatty Acids
SD	Standard Deviation
SPM	Specialized Pro-resolving Mediators
SSL	Semi-Supervised Learning
STHAM	Smart Technologies for Healthy Ageing with Multimorbidity (project acronym)
SVM	Support Vector Machine
TNF	Tumor Necrosis Factor
TRIF	TIR-domain-containing Adapter-Inducing Interferon-β
WGCNA	Weighted Gene Co-expression Network Analysis
